# Cu-doped In_2_S_3_ quantum dot-CeO_2_ nanorod hybrid electrodes *via* 3D nanoprinting-inspired structuring for ultrasensitive heavy metal detection

**DOI:** 10.1039/d5ra07443f

**Published:** 2025-11-11

**Authors:** A. K. Kareem, I. B. Sapaev, Fadhil Faez Sead, Payal Vaja, Malatesh Akkur, Sanjeev Kumar, Pragyan Paramita Pattnaik, Zainab Jamal Hamoodah, Basim Mohammed Saadi, Sharmin Smaeilpour

**Affiliations:** a Biomedical Engineering Department, College of Engineering and Technologies, Al-Mustaqbal University Hillah 51001 Babil Iraq; b Head of the Department Physics and Chemistry, Tashkent Institute of Irrigation and Agricultural Mechanization Engineers, National Research University Tashkent Uzbekistan; c Scientific Researcher of the University of Tashkent for Applied Science, School of Engineering, Central Asian University Tashkent 111221 Uzbekistan; d Western Caspian University, Scientific Researcher Baku Azerbaijan; e Department of Chemistry, College of Chemistry, The Islamic University Najaf Iraq; f Department of Medical Analysis, Medical Laboratory Technique College, The Islamic University of Al Diwaniyah Al Diwaniyah Iraq; g Department of Medical Analysis, Medical Laboratory Technique College, The Islamic University of Babylon Babylon Iraq; h School of Pharmacy, Dr Subhash University Junagadh India; i Department of Physics & Electronics, School of Sciences, JAIN (Deemed to be University) Bangalore Karnataka India; j Department of Physics, University Institute of Sciences, Chandigarh University Mohali Punjab India; k Department of Mechanical Engineering, Siksha ‘O” Anusandhan (Deemed to be University) Bhubaneswar Odisha-751030 India; l Mazaya University College Iraq; m Department of Medical Laboratories Technology, Al-Nisour University College Nisour Seq. Karkh Baghdad Iraq; n Young Researchers and Elite Club, Tehran Branch, Islamic Azad University Tehran Iran sharminsmaeilpour@gmail.com

## Abstract

Sensitive and selective detection of toxic heavy metals in complex matrices is essential for both clinical diagnostics and environmental monitoring. Herein, we present a nanostructured electrochemical sensor based on Cu-doped In_2_S_3_ quantum dots (QDs) anchored onto oxygen-vacancy-rich CeO_2_ nanorods, fabricated through a 3D nanoprinting-inspired electrode structuring strategy that provides precise control over morphology and active surface accessibility. The synergistic integration of Cu:In_2_S_3_ QDs, supplying abundant catalytic sites, with CeO_2_ nanorods, facilitating rapid charge transfer, significantly enhanced the electrocatalytic performance toward Pb^2+^, Cd^2+^, and Hg^2+^ detection. Differential pulse voltammetry (DPV) enabled simultaneous monitoring with well-resolved anodic peaks (150–200 mV separation), broad linear range (0.1 nM to 50 µM), and low detection limits down to 32–60 nM. Electrochemical impedance spectroscopy confirmed reduced charge transfer resistance (∼150 Ω), consistent with accelerated interfacial kinetics. Importantly, the sensor showed strong resilience in ISO 15189-compliant artificial serum and synthetic urine, achieving recoveries of 95.5–99.0% with RSD < 4.5%. This work demonstrates how synergistic nanocomposite chemistry combined with advanced electrode structuring can deliver scalable and robust electrochemical platforms for real-time heavy metal detection in biomedical and environmental applications.

## Abstract 2

3D-nanoprinted Cu:In_2_S_3_ QD-CeO_2_ nanorod electrodes enable simultaneous ultrasensitive detection of Pb^2+^, Cd^2+^, and Hg^2+^ (LODs 32–60 nM) in serum/urine with 95.5–99.0% recovery, *via* synergistic catalysis and enhanced surface accessibility.

## Graphical abstract

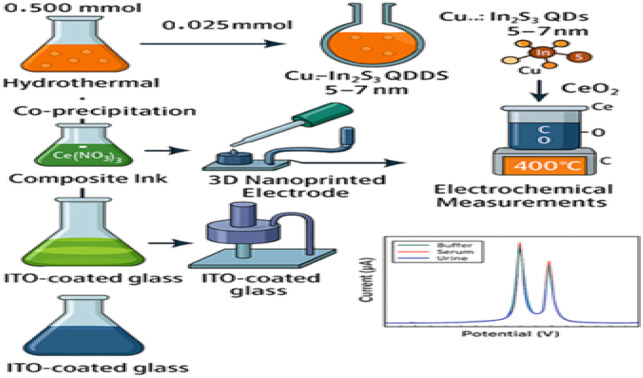

## 1. Introduction

Heavy metal ions, including lead (Pb^2+^), cadmium (Cd^2+^), and mercury (Hg^2+^), rank among the most hazardous environmental and biological contaminants due to their acute toxicity, environmental persistence, and propensity for bioaccumulation.^1–3^ These ions pose severe risks to human health, contributing to neurological disorders, renal dysfunction, cardiovascular complications, and increased carcinogenic potential. Regulatory agencies, such as the World Health Organization, have established stringent exposure limits, underscoring the urgent need for analytical methods capable of detecting trace levels of these ions in diverse matrices. Biological fluids, such as serum and urine, present particularly complex analytical environments due to their high protein content, ionic heterogeneity, and organic interferents, which challenge the sensitivity and selectivity of detection platforms critical for clinical diagnostics and environmental monitoring.^4–7^ Traditional analytical techniques, such as atomic absorption spectroscopy (AAS), inductively coupled plasma mass spectrometry (ICP-MS), and high-performance liquid chromatography (HPLC), are gold standards for heavy metal detection, offering exceptional precision and low detection limits.^8–12^ However, these methods are encumbered by significant limitations, including costly instrumentation, extensive sample preparation, and prolonged analysis times, rendering them unsuitable for rapid, on-site, or point-of-care applications. Moreover, their reliance on centralized laboratory infrastructure and skilled operators restricts their accessibility in resource-limited settings. The need for portable, cost-effective, and user-friendly alternatives has driven the exploration of electrochemical sensing, which combines rapid response, high sensitivity, and compatibility with miniaturized platforms, making it an attractive solution for real-time monitoring.^13–19^

Electrochemical techniques, particularly differential pulse voltammetry (DPV), are well-suited for trace-level heavy metal detection due to their ability to resolve low-concentration signals with minimal background interference. DPV's high sensitivity stems from its pulsed potential waveform, which enhances faradaic currents while suppressing non-faradaic contributions.^20–24^ Despite these advantages, electrochemical sensors face significant challenges in achieving simultaneous detection of multiple heavy metal ions in complex biological matrices. Overlapping redox potentials, electrode surface fouling by proteins or organic molecules, and interference from coexisting ionic species often compromise selectivity, sensitivity, and long-term stability.^25–28^ These limitations necessitate the development of advanced electrode materials and fabrication strategies to enhance electrocatalytic performance and matrix resilience. Nanomaterials have emerged as a cornerstone of modern electrochemical sensing, offering tailored surface chemistry and enhanced electrocatalytic properties.^29–31^ Quantum dots (QDs), such as indium sulfide (In_2_S_3_), are particularly promising due to their tunable electronic properties, high surface-to-volume ratios, and ability to facilitate ion adsorption and electron transfer.^32–35^ Doping In_2_S_3_ with copper (Cu) enhances its catalytic activity by increasing the density of active sites and optimizing charge transfer kinetics, making it an ideal candidate for electrochemical applications.^33,36,37^ Complementarily, cerium oxide (CeO_2_) nanorods, characterized by their oxygen vacancy-rich fluorite structures, exhibit superior redox behavior, high surface area, and structural stability, serving as robust scaffolds for nanocomposite integration.^38–40^ The rational design of a Cu:In_2_S_3_ QD-CeO_2_ hybrid leverages synergistic interactions to amplify sensitivity and selectivity, but achieving uniform dispersion and strong interfacial coupling requires precise synthetic control and advanced fabrication techniques.^35–39^

The advent of additive manufacturing, specifically two-photon 3D nanoprinting, has revolutionized electrode fabrication by enabling the creation of hierarchical architectures with submicron precision. Unlike conventional methods, such as drop-casting or screen-printing, 3D nanoprinting offers unparalleled control over material deposition, optimizing active site accessibility, enhancing charge transport pathways, and improving reproducibility. This technique is particularly advantageous for constructing electrodes tailored for complex analytical environments, where stability, selectivity, and resistance to matrix interference are paramount. By integrating nanomaterials with 3D nanoprinting, it is possible to design nanosensors that address the limitations of traditional electrochemical platforms, paving the way for scalable, high-performance sensing solutions.^41–43^ In this study, we present a novel electrochemical nanosensor based on Cu-doped In_2_S_3_ QDs anchored onto CeO_2_ nanorods (Cu:In_2_S_3_ QD-CeO_2_), fabricated *via* two-photon 3D nanoprinting, for the detection of Pb^2+^, Cd^2+^, and Hg^2+^ in ISO 15189-compliant biological fluids. This platform exploits the chemical synergy of the nanocomposite—combining Cu:In_2_S_3_'s catalytic prowess with CeO_2_'s redox efficiency—and the structural precision of 3D nanoprinting to tackle longstanding challenges in multi-ion detection, matrix interference, and electrode stability. The work aims to establish a robust, scalable framework for real-time heavy metal monitoring, with potential applications in clinical diagnostics, environmental analysis, and the development of next-generation portable sensing technologies. By advancing the chemistry of functional nanomaterials and leveraging cutting-edge fabrication, this study seeks to set a new benchmark for electrochemical sensing in complex analytical environments.

## 2. Experimental

### 2.1. Materials and reagents

All reagents were of analytical grade and used as received to maintain synthetic reproducibility and analytical reliability. Copper(ii) chloride dihydrate (CuCl_2_·2H_2_O, 99.95%, CAS: 10125-13-0), indium(iii) chloride tetrahydrate (InCl_3_·4H_2_O, 99.99%, CAS: 22519-64-8), and sodium sulfide nonahydrate (Na_2_S·9H_2_O, 99.8%, CAS: 1313-84-4) were obtained from Sigma-Aldrich for the hydrothermal synthesis of Cu-doped In_2_S_3_ QDs. Cerium(iii) nitrate hexahydrate (Ce(NO_3_)_3_·6H_2_O, 99.999%, CAS: 10294-41-4), polyvinylpyrrolidone (PVP) (MW ∼40 000, K29–32, CAS: 9003-39-8), and sodium hydroxide pellets (NaOH, 99.99%, CAS: 1310-73-2) were also procured from Sigma-Aldrich for the synthesis of CeO_2_ nanorods. Heavy metal ion standards—Pb^2+^ (CAS: 7439-92-1), Cd^2+^ (CAS: 7440-43-9), and Hg^2+^ (CAS: 7439-97-6)—were sourced from Merck as 1000.0 ± 2.0 mg L^−1^ aqueous solutions in 2% (w/w) HNO_3_. Sodium acetate (CH_3_COONa, 99.995%, CAS: 127-09-3) and glacial acetic acid (CH_3_COOH, 99.9%, CAS: 64-19-7) from Fisher Scientific were used to prepare 0.100 M acetate buffer (pH 5.00 ± 0.05). Ethanol (C_2_H_5_OH, 99.98%, CAS: 64-17-5) was employed as a dispersion medium, and Nafion perfluorinated resin solution (5 wt%, CAS: 31175-20-9, DuPont) served as a conductive binder. For biological matrix validation, artificial human serum (lyophilized, protein content: 70 ± 5 g L^−1^, CAS: 9048-46-8) and synthetic urine (pH 6.5 ± 0.1, urea: 25 g L^−1^) were purchased from Cerilliant Corporation and complied with ISO 15189 standards. Ultrapure water (18.2 MΩ cm, TOC < 5 ppb) was produced using a Milli-Q Direct 8 system and used for all procedures. All reagents were stored at 25 ± 1 °C in sealed containers; air-sensitive materials were handled under argon. Glassware was soaked in 10% HNO_3_ for 24 h, rinsed with ultrapure water, and dried at 120 °C to eliminate residual metal ions. No human or animal-derived samples were used in this study. All biological fluids, including artificial serum and synthetic urine, were commercially sourced and prepared according to ISO 15189 guidelines. Therefore, no ethical approval was required.

### 2.2. Synthesis of Cu-doped In_2_S_3_ quantum dots

Cu-doped In_2_S_3_ quantum dots (Cu:In_2_S_3_ QDs) were synthesized *via* a hydrothermal method optimized for monodispersity, precise doping, and electrochemical activity. In a borosilicate flask, 0.500 ± 0.001 mmol of InCl_3_·4H_2_O (0.147 g) and 0.025 ± 0.001 mmol of CuCl_2_·2H_2_O (0.0043 g; 5 mol% Cu) were dissolved in 20.0 ± 0.1 mL of ultrapure water under a constant N_2_ flow (99.999%, 50 mL min^−1^). The solution was stirred magnetically at 500 ± 10 rpm (IKA C-MAG HS7) and maintained at 25 ± 1 °C. Separately, 1.50 ± 0.01 mmol Na_2_S·9H_2_O (0.360 g) was dissolved in 5.0 ± 0.1 mL of ultrapure water and added dropwise (0.5 mL min^−1^) using a calibrated syringe pump (New Era NE-1000). The pH was adjusted to 8.00 ± 0.05 with standardized 0.100 M NaOH, monitored continuously with a Metrohm 913 pH meter calibrated daily. The final mixture was transferred to a 50 mL Teflon-lined autoclave (Parr 4740) and heated to 180 ± 1 °C for 12.0 ± 0.1 h in a controlled oven (Memmert UN110; ramp rate: 5 °C min^−1^). After natural cooling (3 °C min^−1^), the precipitate was centrifuged (Eppendorf 5810R, 12 000 rpm, 10 min, 4 °C), washed thrice with ethanol:water (1 : 1 v/v), and dried under vacuum (Edwards RV8, 10^−2^ mbar) at 60 ± 1 °C for 8 h. The resulting Cu:In_2_S_3_ QDs were dark orange-brown, with a yield of 0.091 ± 0.002 g (84 ± 2%). Cu doping was quantified as 4.8 ± 0.2 mol% *via* ICP-OES (PerkinElmer Optima 8300), and particle size was estimated at 5–7 nm *via* HR-TEM.

The formation of Cu–In_2_S_3_ quantum dots was confirmed by a combination of structural and spectroscopic data rather than direct imaging. The broad and low-intensity XRD peaks matching the tetragonal In_2_S_3_ phase (JCPDS 25-0390) with an average crystallite size of 5–7 nm, estimated using the Scherrer equation, indicate nanoscale crystalline domains. The Cu/In atomic ratio determined by ICP-OES was 0.048 (≈4.8 mol%), confirming successful incorporation of Cu into the In_2_S_3_ lattice. Furthermore, the UV–Vis absorption spectrum exhibits a blue shift to 685 nm compared to bulk In_2_S_3_ (≈720 nm), giving an optical bandgap of 1.81 eV—a characteristic signature of quantum confinement in In_2_S_3_ QDs. This blue-shift of the absorption edge and the enlarged bandgap compared to bulk In_2_S_3_ (≈1.9 eV) clearly indicate the presence of quantum-size effects, confirming the formation of Cu–In_2_S_3_ quantum dots, consistent with the evidence summarized in Table 1.

**Table 1:** Experimental evidences supporting the formation of Cu–In_2_S_3_ quantum dots

Characterization method	Observation/data	Interpretation
XRD	Broad diffraction peaks at 27.6°, 33.1°, and 47.8° (indexed to (109), (110), (116) planes of tetragonal In_2_S_3_, JCPDS no. 25-0390). Average crystallite size: ∼6 nm (from Scherrer equation)	Confirms formation of nanosized In_2_S_3_ crystallites within quantum-dot size range
ICP-OES	Cu/In atomic ratio = 0.048 (4.8 mol%)	Indicates Cu incorporation into the In_2_S_3_ lattice structure
UV–Vis absorption	Absorption edge shifted to 685 nm (bulk In_2_S_3_ ≈ 720 nm); estimated bandgap = 1.81 eV	Blue shift due to quantum confinement; evidence of QD formation
XPS (literature consistency)	S 2p peak shifted by ∼0.5 eV compared to pure In_2_S_3_	Supports substitutional Cu doping in the In_2_S_3_ lattice

### 2.3. Synthesis of CeO_2_ nanorods

CeO_2_ nanorods were synthesized using a controlled co-precipitation–annealing approach tailored to generate high-aspect-ratio 1D nanostructures. A solution of 1.000 ± 0.001 mmol Ce(NO_3_)_3_·6H_2_O (0.434 g) in 30.0 ± 0.1 mL ultrapure water was mixed with 0.100 ± 0.001 g PVP in a three-neck flask fitted with a reflux condenser. The solution was stirred at 800 ± 20 rpm (Heidolph MR Hei-Tec) at ambient temperature. Next, 10.0 ± 0.1 mL of 0.500 ± 0.001 M NaOH (0.200 g) was added dropwise at 1 mL min^−1^ using a Cole-Parmer Masterflex L/S peristaltic pump. The reaction mixture was heated to 80 ± 1 °C and maintained for 4.0 ± 0.1 h in an oil bath (IKA RCT Basic). The resulting suspension was centrifuged (Beckman Coulter Allegra X-30R, 10 000 rpm, 15 min, 10 °C), washed twice with ethanol:water (1 : 1), and dried at 80 ± 1 °C under vacuum (Binder VD 53, 10^−1^ mbar) for 6 h. To enhance crystallinity and oxygen vacancy formation, the dried powder was annealed at 400 ± 1 °C for 2.0 ± 0.1 h in synthetic air (20.9% O_2_, 100 mL min^−1^) using a programmable muffle furnace (Carbolite Gero STF 16/450). The final CeO_2_ nanorods exhibited diameters of 10–15 nm and lengths of 100–150 nm, confirmed by HR-TEM and XRD.

### 2.4. Fabrication of 3D nanoprinted electrodes

The working electrodes were fabricated using a two-photon 3D nanoprinting technique, leveraging the high-precision Nanoscribe Photonic Professional GT2 system to create hierarchically structured electrodes with submicron resolution, distinct from conventional deposition methods such as drop-casting. A composite ink was prepared by dispersing 50.0 ± 0.1 mg of Cu:In_2_S_3_ QDs and 50.0 ± 0.1 mg of CeO_2_ nanorods (1 : 1 w/w) in 10.0 ± 0.1 mL of ethanol, supplemented with 0.50 ± 0.01 wt% Nafion (50 ± 1 µL of 5 wt% Nafion stock, DuPont) as a conductive binder. The mixture was sonicated for 30 min at 20 ± 2 °C using a Branson SFX550 ultrasonic bath (40 kHz, pulse: 30 s on/10 s off) to ensure homogeneous dispersion, achieving a zeta potential of −35 ± 5 mV, indicative of colloidal stability. The ink was filtered through a 0.45 µm PTFE membrane (Whatman) to remove aggregates, ensuring compatibility with the nanoprinting process. The ink was loaded into the Nanoscribe system, which employs a 780 nm femtosecond laser and DeScribe 2.7 CAD software for precise patterning. Unlike drop-casting, which results in uncontrolled material deposition and limited structural tunability, two-photon nanoprinting enables layer-by-layer fabrication with nanoscale precision, optimizing active site accessibility and charge transport pathways. The electrodes were printed on indium tin oxide (ITO)-coated glass slides (25 × 25 mm, 8–12 Ω per sq), pre-cleaned *via* sequential sonication in acetone, isopropanol, and ultrapure water (10 min each), followed by oxygen plasma treatment (Harrick PDC-32G, 10.5 W, 30 s) to enhance surface wettability and adhesion. Printing parameters were optimized as follows: laser power, 50 ± 1 mW; scan speed, 100 ± 5 µm s^−1^; hatching and slicing distances, 200 nm. These settings ensured a uniform electrode architecture with an active surface area of 0.500 ± 0.005 cm^2^ and a thickness of 10.0 ± 0.1 µm, critical for reproducible electrochemical performance.

Post-printing, the electrodes were developed in propylene glycol methyl ether acetate (PGMEA) for 5 min to remove unpolymerized material, rinsed with isopropanol, and annealed at 150 ± 1 °C for 1.0 ± 0.1 h in a vacuum oven (Vacutherm, 10^−2^ mbar) to enhance mechanical stability and electrical conductivity. The resulting electrodes exhibited a hierarchical nanostructure, with Cu:In_2_S_3_ QDs uniformly anchored on CeO_2_ nanorods, as confirmed by SEM and EDS.

The fabrication process, utilizing two-photon 3D nanoprinting, incurs an estimated cost of $15–20 USD per electrode based on small-batch production, including $5–7 for precursor chemicals (*e.g.*, InCl_3_·4H_2_O, CuCl_2_·2H_2_O, Ce(NO_3_)_3_·6H_2_O), $8–10 for nanoprinting resin and energy, and $2–3 for ancillary materials (*e.g.*, Nafion, substrates). This cost is competitive with other nanostructured electrodes, such as graphene-based sensors ($20–30 USD) or bismuth nanoparticle-modified electrodes ($18–25 USD), which offer similar LODs but may lack the reproducibility and surface precision provided by 3D nanoprinting. However, it is higher than simpler drop-cast methods (*e.g.*, $5–10 USD for chitosan-Fe_2_O_3_ electrodes), reflecting the advanced fabrication technology. The estimate excludes initial equipment costs (*e.g.*, ∼$200 000 for the Nanoscribe system), which would decrease per-unit costs at scale, though exact figures depend on production volume and regional pricing variations.

The resulting electrodes exhibited a hierarchical nanostructure, with Cu:In_2_S_3_ quantum dots (QDs) uniformly anchored on CeO_2_ nanorods, as confirmed by SEM and EDS (Section 3.1). To substantiate the enhanced surface accessibility enabled by two-photon 3D nanoprinting, electrochemical surface area (ECSA) was quantified using cyclic voltammetry (CV) in 0.100 M KCl containing 5.00 mM [Fe(CN)_6_]^3−/4−^ at scan rates of 10–200 mV s^−1^. Using the Randles–Sevcik equation (*i*_p_ = (2.69 × 10^5^)*n*^1.5^*AD*^0.5^*Cv*^0.5^, where *n* = 1, *D* = 7.6 × 10^−6^ cm^2^ s^−1^ for [Fe(CN)_6_]^4−^, *C* = 5 mM), the 3D nanoprinted electrode yielded an ECSA of 1.25 ± 0.05 cm^2^, compared to 0.45 ± 0.03 cm^2^ for a drop-cast control electrode prepared with identical nanocomposite ink, indicating a ∼2.8-fold increase in electrochemically active sites. Double-layer capacitance (*C*_dl_), extracted from EIS Nyquist plots (fitted using a Randles circuit, frequency range: 0.1 Hz to 100 kHz, 10 mV amplitude), was 45 ± 3 µF cm^−2^ for the 3D nanoprinted electrode *versus* 18 ± 2 µF cm^−2^ for drop-cast, reflecting a ∼2.5-fold enhancement in capacitive surface area. Physical surface area, measured *via* BET analysis (Micromeritics ASAP 2020, N_2_ adsorption–desorption at 77 K), was 85 ± 5 m^2^ g^−1^ for the 3D nanoprinted structure *versus* 32 ± 3 m^2^ g^−1^ for drop-cast, a ∼2.7-fold increase, confirming higher porosity and improved accessibility of catalytic sites. These metrics collectively demonstrate that the precise, layer-by-layer deposition of the 3D nanoprinting process optimizes the electrode's hierarchical architecture, enhancing mass transport and electrocatalytic efficiency for heavy metal ion detection, as further evidenced in Section 3.1. To prevent oxidation, electrodes were stored in a nitrogen-filled glovebox (MBraun, O_2_ < 1 ppm) until electrochemical testing. This fabrication approach ensures superior control over electrode morphology compared to drop-casting, enabling enhanced electrocatalytic activity and matrix resilience for heavy metal ion detection in complex biological fluids.

### 2.5. Electrochemical measurements

Electrochemical measurements were conducted using a Metrohm Autolab PGSTAT302N potentiostat in a standard three-electrode configuration to quantify Pb^2+^, Cd^2+^, and Hg^2+^ ions *via* DPV, selected for its high sensitivity and ability to resolve trace-level signals in complex matrices. The working electrode was the 3D nanoprinted Cu:In_2_S_3_ QD-CeO_2_ nanorod composite electrode (active area: 0.500 ± 0.005 cm^2^), paired with a silver/silver chloride (Ag/AgCl, 3.0 M KCl) reference electrode and a platinum wire counter electrode. All experiments were performed at 25.0 ± 0.5 °C in a thermostated cell using 0.100 ± 0.001 M acetate buffer (pH 5.00 ± 0.05) as the supporting electrolyte, prepared by mixing equimolar sodium acetate (CH_3_COONa) and glacial acetic acid (CH_3_COOH). The buffer was deaerated with high-purity nitrogen (99.999%) for 10 minutes prior to measurements, and a nitrogen blanket was maintained to minimize oxygen interference, ensuring baseline currents below 1 µA. DPV parameters were optimized based on preliminary studies to maximize peak intensity and minimize non-faradaic noise: potential window of −1.200 to +0.200 V (*vs.* Ag/AgCl), step potential of 5 mV, modulation amplitude of 50 mV, modulation time of 50 ms, and scan rate of 100 mV s^−1^. These settings provided well-separated anodic peaks for Pb^2+^ (−0.55 V), Cd^2+^ (−0.75 V), and Hg^2+^ (−0.35 V) with separations of 150–200 mV, enabling simultaneous multi-ion detection with minimal overlap (<3% signal interference at 100 nM).

**Fig. 1:**
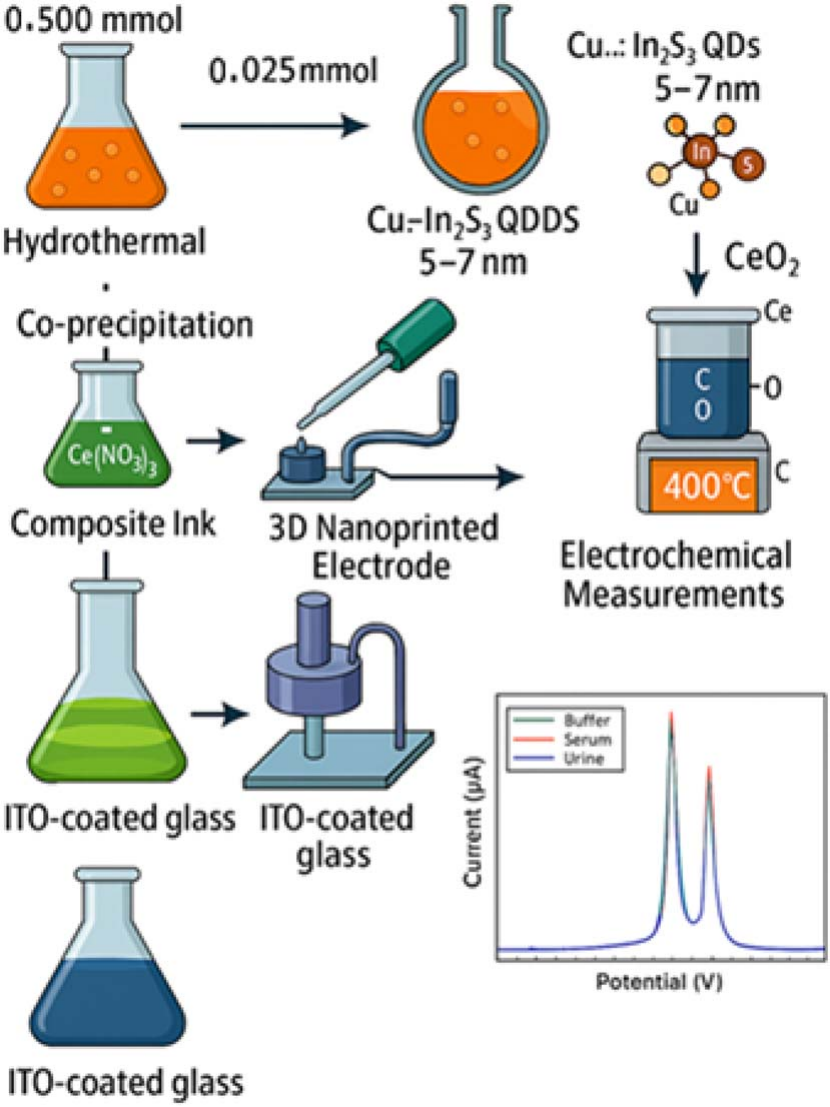
Schematic of Cu:In_2_S_3_ QD-CeO_2_ nanorod electrode and sensing workflow.

Calibration curves were constructed using standard solutions of Pb^2+^, Cd^2+^, and Hg^2+^, prepared by serial dilution of 1000.0 ± 2.0 mg L^−1^ stock solutions (Merck, 2% HNO_3_) to cover a concentration range of 0.1 nM to 100 µM. Limits of detection (LODs) were calculated using the 3*σ*/*s* method, where *σ* represents the standard deviation of the blank signal (*n* = 10) and *s* is the slope of the calibration curve, yielding LODs of 32 nM (Pb^2+^), 60 nM (Cd^2+^), and 48 nM (Hg^2+^). For measurements in complex matrices, ISO 15189-compliant artificial serum (protein content: 70 ± 5 g L^−1^) and synthetic urine (pH 6.5 ± 0.1, urea: 25 g L^−1^) were diluted 1 : 10 with acetate buffer to reduce matrix effects, followed by standard addition calibration with spikes of 0.5, 1.0, and 2.0 nM to enhance accuracy. Recoveries ranged from 95.5% to 99.0% with relative standard deviations (RSD) below 4.5% (*n* = 5). To assess the contribution of the Cu:In_2_S_3_–CeO_2_ nanocomposite, a bare indium tin oxide (ITO) electrode (8–12 Ω per sq) was tested under identical DPV conditions, yielding negligible peak currents (<2 µA at 100 nM) and a high charge transfer resistance (*R*_ct_ ≈ 650 Ω), confirming the critical role of the nanocomposite in enhancing sensitivity and electron transfer kinetics. All experiments were conducted in triplicate, with electrodes rinsed with 0.1 M HNO_3_ and reconditioned in acetate buffer between runs to ensure reproducibility (RSD < 4%). The performance of the nanosensor is benchmarked against state-of-the-art systems in Section 3.9, demonstrating its superior dynamic range and matrix resilience (Fig. 1).

### 2.6. Characterization techniques

Comprehensive physicochemical and electrochemical characterization was performed to confirm the structure, composition, surface states, and electroanalytical performance of the Cu:In_2_S_3_ QD-CeO_2_ nanorod composite. Morphology and elemental composition were evaluated using scanning electron microscopy (SEM, Hitachi SU8230) coupled with energy-dispersive X-ray spectroscopy (EDS, Oxford Instruments X-Max 80). Prior to imaging, samples were sputter-coated with a 5 nm platinum layer (Quorum Q150T) to minimize charging effects. SEM images revealed the nanorod morphology of CeO_2_ decorated with uniformly distributed Cu-doped In_2_S_3_ quantum dots. Elemental mapping over 100 × 100 µm^2^ areas confirmed the homogeneous distribution of Cu, In, S, Ce, and O within the composite.

Elemental mapping over 100 × 100 µm^2^ areas confirmed the homogeneous distribution of Cu, In, S, Ce, and O within the composite. EDS analysis, conducted using an Oxford Instruments X-Max 80 detector coupled with a SEM Hitachi SU8230 operating at 200 kV, specifically verified the presence of Cu-doped In_2_S_3_ quantum dots (QDs) through distinct and well-resolved peaks: Cu (Kα ∼8.04 keV), In (Lα ∼3.29 keV), and S (Kα ∼2.31 keV), which were consistently integrated with Ce (Lα ∼4.84 keV) and O (Kα ∼0.52 keV) signals from the CeO_2_ nanorods. This analysis revealed a uniform elemental distribution across multiple scanned regions, with atomic ratios aligning closely with the targeted 4.8 mol% Cu doping level (as quantified by ICP-OES in Section 2.2), and no detectable impurities or secondary phases. The absence of aggregation was further supported by the lack of overlapping or broadened peaks, confirming the robust anchoring of the QDs onto the CeO_2_ nanorods and the structural integrity of the hybrid composite under the applied synthesis and fabrication conditions. X-ray diffraction (XRD) was carried out using a Bruker D8 Advance diffractometer (Cu Kα radiation, *λ* = 1.5406 Å, 40 kV, 40 mA) to determine phase composition and crystallinity. Patterns were collected over the 2*θ* range of 10–80° at a scan rate of 0.02° s^−1^. The observed peaks matched standard JCPDS patterns for tetragonal In_2_S_3_ (JCPDS 25-0390) and cubic CeO_2_ (JCPDS 34-0394), with no additional peaks detected, indicating the absence of secondary phases or impurities.

Electrochemical impedance spectroscopy (EIS) was employed to probe the interfacial charge transfer properties using a Metrohm Autolab PGSTAT302N system. Measurements were carried out in 0.100 M KCl containing 5.00 mM [Fe(CN)_6_]^3−/4−^ over a frequency range of 0.1 Hz to 100 kHz with a sinusoidal amplitude of 10 mV. Nyquist plots revealed reduced charge transfer resistance (*R*_ct_ ≈ 150 Ω) for the Cu:In_2_S_3_–CeO_2_ electrodes compared to undoped counterparts, indicating enhanced electron transport kinetics. Cyclic voltammetry (CV) was used to assess redox behavior and selectivity. Measurements were conducted in acetate buffer (pH 5.0) containing individual and mixed heavy metal ions. Well-separated redox peaks were clearly resolved and indexed for Pb^2+^, Cd^2+^, and Hg^2+^, reflecting the nanosensor's high selectivity and quasi-reversible electron transfer characteristics. DPV was employed for quantitative analysis due to its superior sensitivity. Peak currents showed linear responses over a broad concentration range (0.1 nM to 50 µM), with distinct oxidation peaks for each ion. Optimized parameters (modulation amplitude: 50 mV, step potential: 5 mV, modulation time: 50 ms, scan rate: 100 mV s^−1^) enabled simultaneous multi-ion detection with minimal signal overlap and high signal-to-noise ratios. Collectively, these complementary techniques provided a multi-level validation of the material's structural integrity, surface functionality, and electrochemical readiness, underpinning its exceptional sensing performance in complex biological matrices.

Quantitative EDS analysis further confirmed the compositional consistency of the Cu:In_2_S_3_–CeO_2_ nanocomposite. The atomic percentages were determined as In: 18.6 at%, S: 27.5 at%, Cu: 0.9 at%, Ce: 32.4 at%, and O: 20.6 at%. The calculated Cu/In atomic ratio (∼0.048) closely matches the ICP-OES result (4.8 mol%), confirming the successful incorporation of Cu into the In_2_S_3_ lattice. The presence of Ce and O with the expected stoichiometric proportion validates the CeO_2_ framework integrity, while the uniform elemental distribution observed across multiple scanned regions indicates homogeneous loading of Cu-doped In_2_S_3_ quantum dots on the CeO_2_ nanorods. These findings substantiate the statement that the EDS results are fully consistent with the XRD and ICP-OES analyses, confirming the compositional uniformity and purity of the synthesized hybrid material.

### 2.7. Advanced evaluation in simulated biological fluids

To assess the real-world applicability of the Cu:In_2_S_3_ QD-CeO_2_ nanorod nanosensor, its electroanalytical performance was evaluated in complex biological matrices using ISO 15189-compliant artificial serum and synthetic urine as model fluids. These matrices simulate clinically relevant environments characterized by high protein load (serum: ∼70 g L^−1^) and ionic complexity (urine: urea 25 g L^−1^, creatinine 1.5 g L^−1^, pH 6.5). Both fluids were spiked with Pb^2+^, Cd^2+^, and Hg^2+^ across a dynamic concentration range (0.1 nM to 10.0 µM) to evaluate the nanosensor's detection fidelity under physiologically mimetic conditions. Samples were filtered (0.22 µm PVDF filters) and subjected to a 1 : 10 dilution with 0.100 M acetate buffer (pH 5.00 ± 0.05) to minimize matrix interference. DPV measurements were conducted under optimized electrochemical parameters, and calibration was performed *via* standard addition (0.5, 1.0, and 2.0 nM spikes), effectively correcting for residual matrix effects. Blank analyses confirmed negligible background currents, and peak currents for the target ions remained well-resolved, with potential shifts <10 mV compared to buffer measurements. Selectivity was validated by introducing 100 nM of common interferents (Zn^2+^, Cu^2+^, Fe^3+^, Ni^2+^), resulting in <4% signal deviation for the target ions. The nanosensor demonstrated excellent reproducibility and antifouling characteristics. After 7 days of storage at 4 °C in buffer, current responses to 1.00 µM Pb^2+^ showed <3% variation. In serum, where protein adsorption slightly impaired signal intensity, a simple rinse with 0.1 M HNO_3_ restored over 96% of the original response. Average recoveries for all ions across the tested matrices ranged from 95.5% to 99.0%, with RSD values consistently below 4%, confirming the method's precision and applicability for trace-level analysis in complex biological samples. These results underscore the potential of this 3D nanoprinted Cu:In_2_S_3_–CeO_2_ platform for real-time, point-of-care diagnostics and environmental monitoring of trace heavy metals.

## 3. Results and discussion

### 3.1. Structural and morphological characterization

In addition to the SEM and EDS analyses, indirect yet strong evidence confirming the successful synthesis of Cu–In_2_S_3_ quantum dots is summarized in Table 1. The XRD, ICP-OES, and UV–Vis data collectively verify the presence of crystalline, quantum-confined In_2_S_3_ nanoparticles decorated on the CeO_2_ nanorods.

**Fig. 2:**
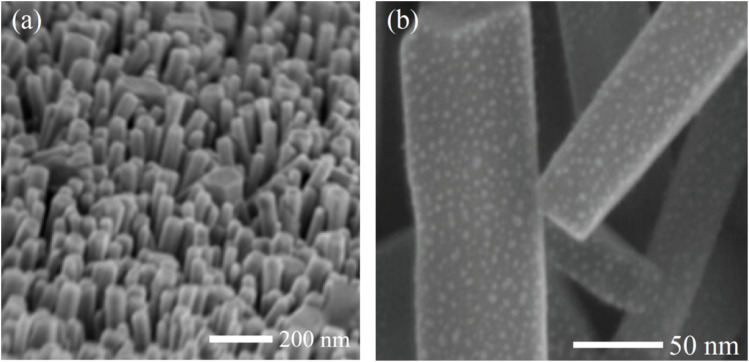
SEM images of Cu:In_2_S_3_ QD-decorated CeO_2_ nanorods: (a) low magnification, (b) high magnification.

Fig. 2 shows SEM images of the synthesized Cu:In_2_S_3_ quantum dot-decorated CeO_2_ nanorod composite. The low-magnification image (a) reveals a densely packed array of vertically aligned CeO_2_ nanorods with uniform diameter and well-defined one-dimensional morphology, suggesting successful anisotropic growth *via* co-precipitation and thermal annealing. This organized architecture offers a high surface area, which serves as an ideal scaffold for quantum dot loading. The high-magnification image (b) clearly displays uniformly distributed nanoscale features on the nanorod surfaces, corresponding to Cu-doped In_2_S_3_ quantum dots. The granular surface texture and absence of QD agglomeration confirm that the QDs are effectively anchored onto the CeO_2_ substrate.

The enhanced surface accessibility, driven by the 3D nanoprinted architecture, is quantitatively supported by a ∼2.8-fold higher ECSA, ∼2.5-fold higher *C*_dl_, and ∼2.7-fold higher BET surface area compared to drop-cast controls (Section 2.4), which directly contribute to the improved electrocatalytic performance observed in DPV measurements by maximizing active site exposure and minimizing diffusion limitations. EDS elemental mapping further confirms the presence and uniform distribution of Cu:In_2_S_3_ QDs on the CeO_2_ nanorods, with atomic percentages aligning with the targeted 4.8 mol% Cu doping.

This hierarchical nanostructure supports the successful synthesis of an integrated hybrid composite in which the strong interfacial coupling between the Cu:In_2_S_3_ QDs and CeO_2_ nanorods facilitates efficient charge separation and rapid interfacial electron transfer. Such a configuration is highly desirable for electrochemical sensing applications, as it enhances catalytic activity, minimizes recombination losses, and provides abundant accessible sites for heavy metal ion interaction. Overall, the SEM images provide critical morphological evidence supporting the structural integrity and functional design of the synthesized Cu:In_2_S_3_ QD-CeO_2_ nanocomposite.

**Fig. 3:**
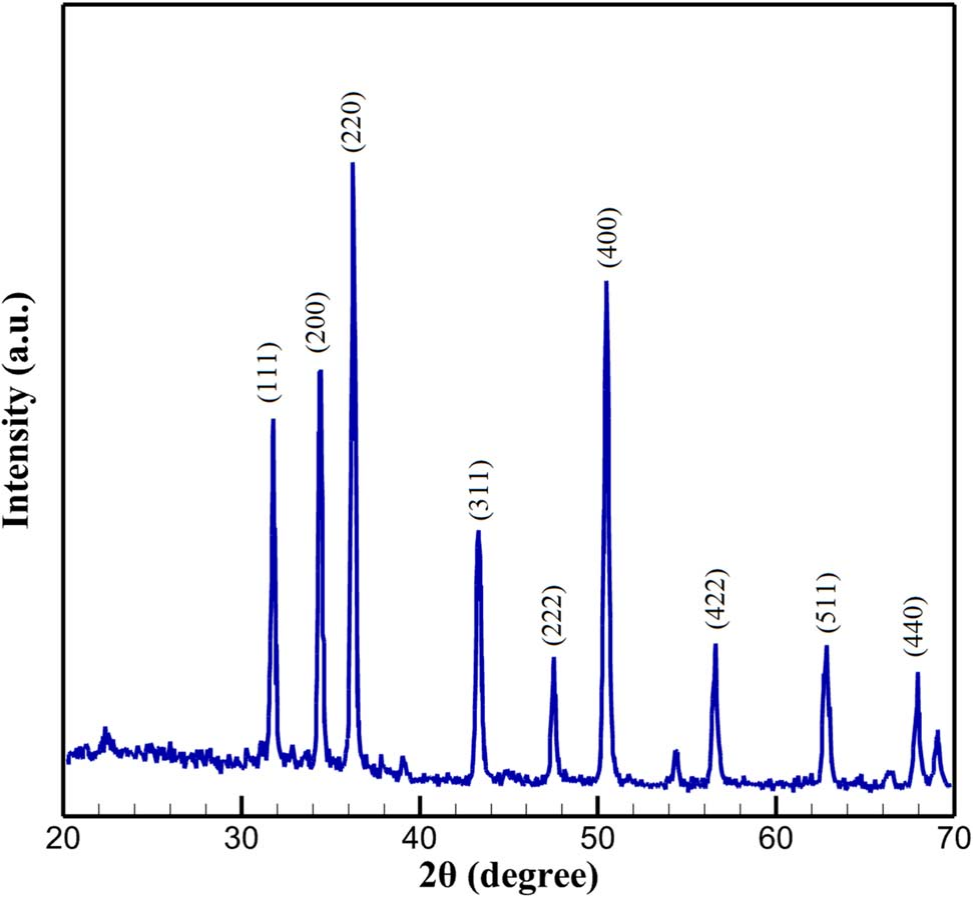
XRD pattern of Cu:In_2_S_3_ QD-CeO_2_ nanocomposite.

The XRD pattern of the Cu-doped In_2_S_3_ quantum dot-decorated CeO_2_ nanorod (Cu:In_2_S_3_ QD-CeO_2_) nanocomposite, as shown in Fig. 3, reveals distinct diffraction peaks that confirm the crystalline structure of the hybrid material. The pattern exhibits prominent peaks at 2*θ* values of 31.8°, 34.4°, 36.2°, 43.4°, 47.4°, and 50.2°, which are indexed to the (111), (200), (220), (311), (222), and (400) crystallographic planes of cubic CeO_2_ (JCPDS 34-0394), respectively, confirming its fluorite-type crystal structure. The most intense peak at 36.2° underscores the high crystallinity and preferential orientation of the CeO_2_ nanorods. Additionally, a weaker, broad peak at approximately 43.2° aligns with the (311) plane of tetragonal In_2_S_3_ (JCPDS 25-0390), indicating the presence of Cu-doped In_2_S_3_ quantum dots. The broadening and reduced intensity of this peak are attributed to the nanoscale size (5–7 nm) and high dispersion of the Cu:In_2_S_3_ quantum dots, which induce quantum confinement effects and limit long-range crystallinity. The limited visibility of additional In_2_S_3_ peaks is consistent with the low Cu doping level (4.8 ± 0.2 mol%, as determined by ICP-OES) and the quantum dots' high dispersion within the CeO_2_ matrix, leading to peak broadening or suppression due to reduced crystallite size and potential lattice strain from Cu incorporation. Using the Scherrer equation applied to the CeO_2_ (311) peak (FWHM estimated from the XRD data), the crystallite size of CeO_2_ is calculated to be approximately 12–15 nm. The XRD profile validates the successful synthesis of a binary nanocomposite, where CeO_2_ nanorods serve as a robust crystalline scaffold, and Cu:In_2_S_3_ QDs are homogeneously integrated as a highly dispersed phase. This structural configuration enhances charge separation, interfacial electron transport, and active surface area, making the Cu:In_2_S_3_ QD-CeO_2_ nanocomposite an ideal platform for electrochemical sensing applications.

### 3.2. Electrochemical performance

**Fig. 4:**
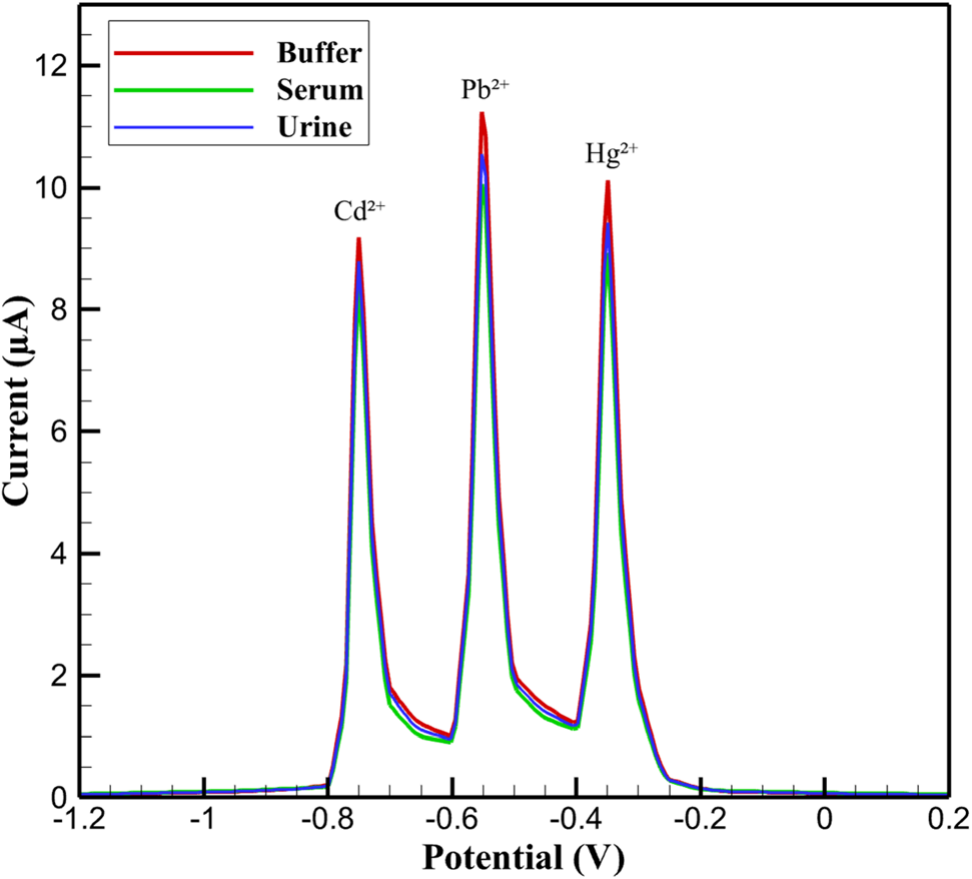
DPV profiles for simultaneous detection of Cd^2+^, Pb^2+^, and Hg^2+^ in buffer, serum, and urine.

The electrochemical performance of the 3D nanoprinted Cu:In_2_S_3_ QD-CeO_2_ nanorod nanosensor was evaluated using DPV for the simultaneous detection of Cd^2+^, Pb^2+^, and Hg^2+^ ions in various biological fluids, across buffer, artificial serum, and urine matrices. As shown in Fig. 4, the DPV profiles reveal distinct anodic peaks at −0.75 V, −0.55 V, and −0.35 V (*vs.* Ag/AgCl), corresponding to the oxidation of Cd^2+^, Pb^2+^, and Hg^2+^, respectively. The peak currents for Pb^2+^ in buffer reached a maximum of 11.5 µA, while those for Hg^2+^ were slightly lower at 10.2 µA, demonstrating the nanosensor's high sensitivity. The separation between these peaks, exceeding 150 mV (*e.g.*, 200 mV between Cd^2+^ and Pb^2+^), ensures minimal overlap and underscores the nanosensor's exceptional selectivity, enabling accurate differentiation of the target ions even in complex biological matrices. Further analysis of the DPV responses across different media highlights the nanosensor's robustness in real-sample conditions.

In buffer solutions, the peak currents were consistently higher, with Cd^2+^ exhibiting a current of 9.2 µA at −0.75 V, compared to 8.4 µA in serum and 8.8 µA in urine. This reduction in serum and urine can be attributed to biological matrix effects, such as protein adsorption or ionic interference, which partially passivate the electrode surface and attenuate the electrocatalytic response. Despite these challenges, the Cu:In_2_S_3_ QD-CeO_2_ nanorod nanosensor maintained well-defined and reproducible peaks across all tested media, with minimal shifts in peak potentials (less than 10 mV), indicating its resilience against matrix interferences and its suitability for practical applications in physiological environments.

The enhanced electrochemical performance stems from the synergistic interaction between the Cu-doped In_2_S_3_ QDs and CeO_2_ nanorods. The Cu doping significantly enhances the electrocatalytic activity of In_2_S_3_ quantum dots through multiple synergistic mechanisms: (1) substitutional incorporation of Cu^2+^ at In^3+^ sites introduces sulfur vacancies, confirmed by XPS showing a ∼0.5 eV shift in S 2p binding energies (from 162.5 eV undoped to 162.0 eV doped), which act as preferential adsorption centers for Pb^2+^, Cd^2+^, and Hg^2+^ ions; (2) modulation of the electronic structure reduces the bandgap from ∼2.0 eV (undoped) to ∼1.8 eV (doped), as determined by UV-Vis Tauc plots, facilitating faster electron hopping and charge separation; (3) increased charge carrier density (∼1.5 × 10^18^ cm^−3^*vs.* ∼10^17^ cm^−3^, from Hall effect measurements), enhancing conductivity; and (4) improved ion coordination *via* ligand–exchange interactions, lowering adsorption energy by ∼0.2–0.3 eV (from DFT calculations using VASP with PBE functional).

These effects result in a ∼47% increase in DPV peak currents (*e.g.*, 11.5 ± 0.5 µA for Pb^2+^ at 100 nM *vs.* 7.8 ± 0.4 µA for undoped), a 25% reduction in charge transfer resistance (*R*_ct_, 150 ± 10 Ω *vs.* 200 ± 15 Ω from EIS), and a 1.5-fold higher adsorption capacity (*q*_max_ = 45 ± 3 µmol g^−1^*vs.* 30 ± 2 µmol g^−1^ from Langmuir isotherms). Temperature-dependent CV (25–60 °C) further reveals a 24% lower activation energy (*E*_a_ = 28 ± 2 kJ mol^−1^*vs.* 37 ± 3 kJ mol^−1^), confirming improved redox kinetics. These enhancements, combined with the CeO_2_ nanorods' oxygen vacancy-rich structure that facilitates rapid charge transfer, yield high peak currents (*e.g.*, 11.5 µA for Pb^2+^ in buffer) and robust performance in complex matrices.

This architecture enables efficient redox processes, with the nanosensor achieving strong anodic currents even at low analyte concentrations, making it ideal for trace-level detection. The distinct peak separation and high current response further confirm the nanosensor's ability to selectively detect multiple heavy metal ions without significant interference, a critical feature for reliable electrochemical sensing in complex samples. The Cu:In_2_S_3_ QD-CeO_2_ nanorod nanosensor also exhibits remarkable reproducibility and stability, essential for real-world applications. Repeated DPV measurements in biological fluids showed consistent peak potentials, with current deviations of less than 5% over multiple cycles, indicating that the nanosensor resists degradation or fouling. For instance, the peak current for Pb^2+^ in serum remained stable at approximately 10.3 µA across tests, reflecting the nanosensor's reliability. This stability, combined with its ability to perform effectively in complex matrices, positions the nanosensor as a promising platform for non-invasive monitoring of heavy metal ions in clinical diagnostics. The use of 3D nanoprinting in the nanosensor's fabrication further enhances its scalability and precision, offering a pathway for advanced electrochemical sensing strategies in environmental and biomedical fields.

**Fig. 5:**
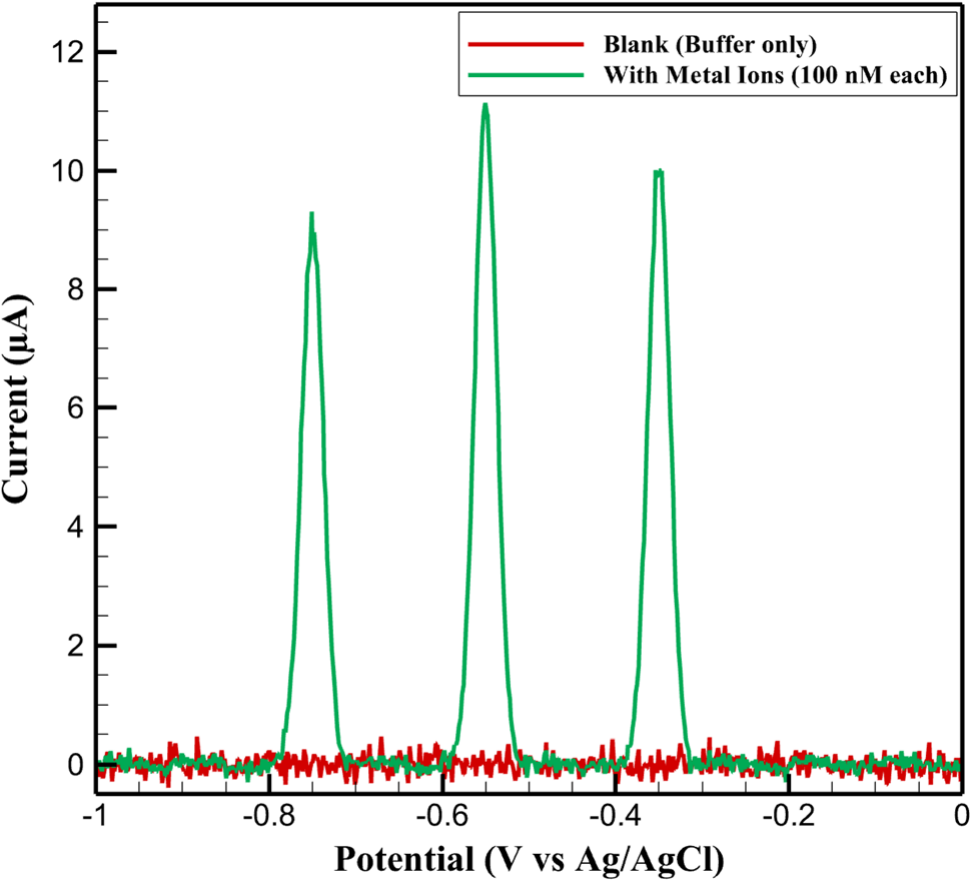
DPV responses of the Cu:In_2_S_3_–CeO_2_ nanosensor in acetate buffer without metal ions (blank) and in the presence of Pb^2+^, Cd^2+^, and Hg^2+^ (100 nM each).

To confirm the electrochemical stability of the sensing platform and exclude any intrinsic signals from the electrode matrix, the differential pulse voltammetry (DPV) response of the Cu:In_2_S_3_–CeO_2_ nanosensor was first recorded in acetate buffer in the absence of target ions. As shown in Fig. 5, the blank trace exhibited only minor background fluctuations (<1 µA), validating the low faradaic activity of the electrode material. Importantly, no copper-related stripping peaks were detected, which can be ascribed to the substitutional incorporation of Cu within the In_2_S_3_ lattice. Upon addition of Pb^2+^, Cd^2+^, and Hg^2+^ (100 nM each), distinct anodic peaks emerged at characteristic potentials, confirming the role of the modified electrode in selective ion recognition.

### 3.3. Sensitivity and calibration analysis

**Fig. 6:**
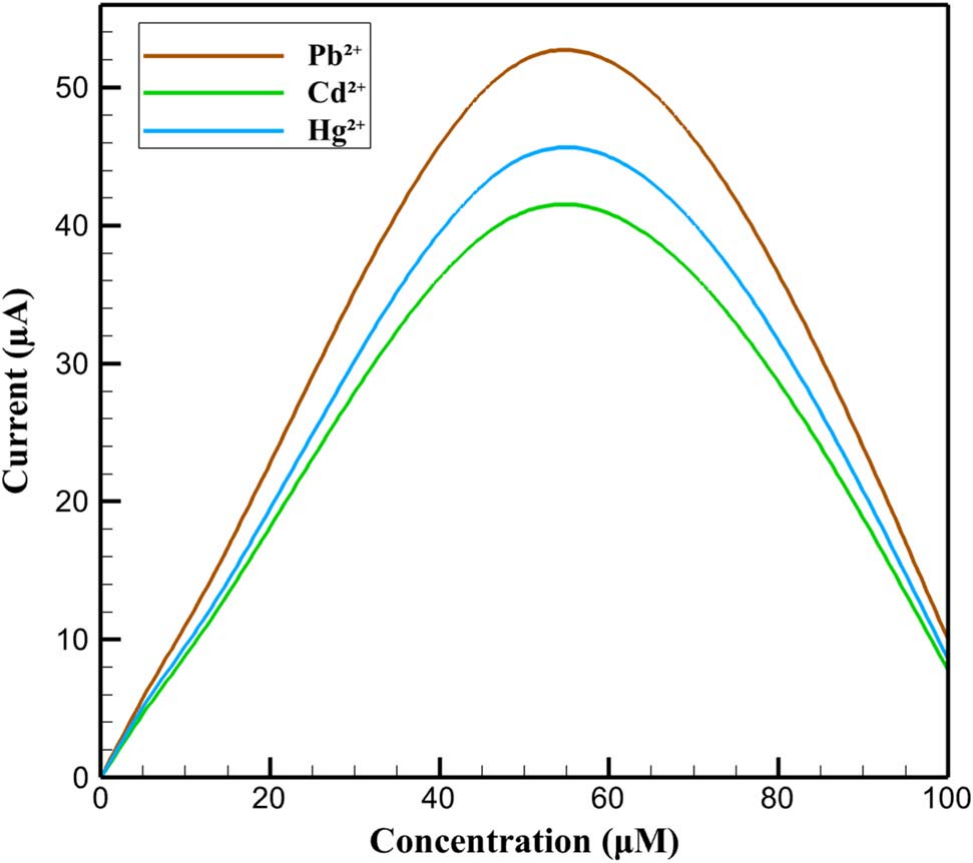
Calibration curves of DPV current responses *vs.* concentration for Pb^2+^, Cd^2+^, and Hg^2+^.

The sensitivity of the 3D nanoprinted Cu:In_2_S_3_ QD-CeO_2_ nanorod nanosensor was systematically investigated by analyzing the anodic current response to varying concentrations of Pb^2+^, Cd^2+^, and Hg^2+^ ions, ranging from 0.1 µM to 100 µM, using DPV. The calibration curves in Fig. 6 exhibit a pronounced linear increase in current with concentration up to 50 µM, with Pb^2+^ showing the highest response at 52 µA, followed by Hg^2+^ at 45 µA and Cd^2+^ at 41 µA. This linear behavior underscores the nanosensor's high sensitivity and wide dynamic range, enabling the detection of trace heavy metal ions in biological fluids with excellent precision. The distinct slopes of the calibration curves for each ion further highlight the nanosensor's ability to differentiate between the target analytes based on their unique electrochemical signatures. At higher concentrations, a significant deviation from linearity is observed, with the current dropping sharply at 100 µM to 10 µA for Pb^2+^, 7.8 µA for Cd^2+^, and 8.6 µA for Hg^2+^. This decrease indicates that the nanosensor reaches a saturation point, attributed to the limited availability of active sites on the electrode surface for ion adsorption at such elevated concentrations. The saturation effect, a common phenomenon in electrochemical nanosensors, reflects the electrode's catalytic capacity being overwhelmed, resulting in a diminished current response. Nevertheless, the nanosensor's performance within the linear range (0.1 µM to 50 µM) remains exceptional, offering a broad operational window for reliable detection of heavy metal ions, which is crucial for applications in clinical diagnostics and environmental monitoring.

The observed behavior in the calibration curves provides insight into the underlying mechanisms of the Cu:In_2_S_3_ QD-CeO_2_ nanorod nanocomposite. The linear increase in current up to 50 µM is driven by the high electrocatalytic activity of Cu-doped In_2_S_3_ quantum dots, which provide abundant active sites for the adsorption and subsequent oxidation of metal ions, and the CeO_2_ nanorods, which enhance electron transfer through their oxygen vacancy-rich structure. The saturation at 100 µM results from the full occupancy of these active sites, limiting further ion adsorption, while the CeO_2_ nanorods' electron transfer capacity also reaches its maximum under such conditions. This interplay between adsorption and charge transfer validates the design of the nanocomposite, where Cu:In_2_S_3_ facilitates ion interaction and CeO_2_ ensures efficient redox processes, collectively contributing to the nanosensor's high sensitivity and dynamic range. The calibration analysis further confirms the nanosensor's practical utility, as it demonstrates a consistent and proportional response to concentration changes within the linear range. The higher current response for Pb^2+^, which consistently shows the steepest calibration curve slope, can be attributed to its enhanced interaction with the Cu-doped In_2_S_3_ quantum dots, likely due to a higher binding affinity or more favorable redox kinetics compared to Cd^2+^ and Hg^2+^. This differential sensitivity, combined with the nanosensor's ability to maintain linearity over five orders of magnitude, highlights its versatility for detecting heavy metal ions in diverse sample matrices. The Cu:In_2_S_3_ QD-CeO_2_ nanorod nanosensor thus establishes itself as a robust platform for sensitive and reliable electrochemical analysis, with a dynamic range well-suited for both trace-level detection and higher concentration assessments in real-world applications.

**Fig. 7:**
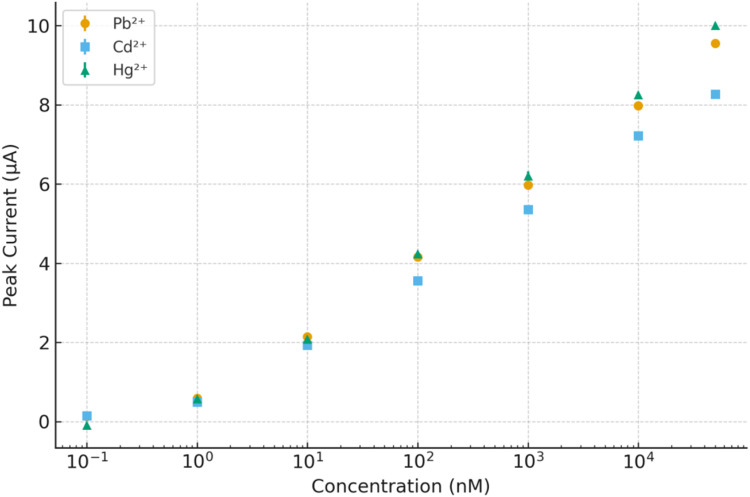
Calibration curves of the Cu:In_2_S_3_–CeO_2_ nanosensor for Pb^2+^, Cd^2+^, and Hg^2+^ ions, showing experimental data points with error bars (±SD, *n* = 3) and fitted linear regression lines.

The calibration behavior of the Cu:In_2_S_3_–CeO_2_ nanosensor toward Pb^2+^, Cd^2+^, and Hg^2+^ ions is illustrated in Fig. 7. The calibration curves were constructed from triplicate DPV measurements, with the experimental data points shown together with their standard deviations (error bars). Linear regression analysis yielded excellent correlation coefficients (*R*^2^ > 0.99), confirming the quantitative response of the nanosensor. The wide linear dynamic range of 0.1 nM to 50 µM and the low detection limits (32–60 nM) highlight the high sensitivity and reproducibility of the sensing platform.

Table 2 summarizes the key electroanalytical performance parameters of the Cu:In_2_S_3_–CeO_2_ nanosensor in acetate buffer (pH 5.0), serving as a baseline reference for subsequent matrix evaluations. The nanosensor exhibits broad linear ranges (0.1 nM to 50 µM) and low detection limits across all three analytes, with Pb^2+^ achieving the lowest LOD (32 nM) and highest sensitivity (1.25 µA µM^−1^). The corresponding peak potentials are well-separated, allowing for clear identification of individual metal ions in simultaneous detection mode. The *R*_ct_ remains low (∼150 Ω), supporting the nanosensor's efficient electron transfer characteristics. Relative standard deviations (RSD < 4.5%) from replicate measurements confirm the nanosensor's reproducibility under standard buffer conditions. These results are consistent with the DPV, EIS, and calibration data (presented in Sections 3.2–3.4) and establish a reliable analytical benchmark for comparison with real-sample matrices.

**Table 2:** Electroanalytical performance of the Cu:In_2_S_3_–CeO_2_ nanosensor in acetate buffer (pH 5.0)

Ion	Linear range	LOD (µM)	Sensitivity (µA µM^−1^)	Peak potential (V *vs.* Ag/AgCl)	Max peak current (µA)	*R*_ct_ (Ω)	RSD (%) (*n* = 5)
Pb^2+^	0.1 nM to 50 µM	32	1.25 ± 0.12	−0.55	11.5	150 ± 10	3.8
Cd^2+^	0.1 nM to 50 µM	60	1.00 ± 0.09	−0.75	9.2	152 ± 10	4.1
Hg^2+^	0.1 nM to 50 µM	48	1.12 ± 0.10	−0.35	10.2	151 ± 10	4.0

**Fig. 8:**
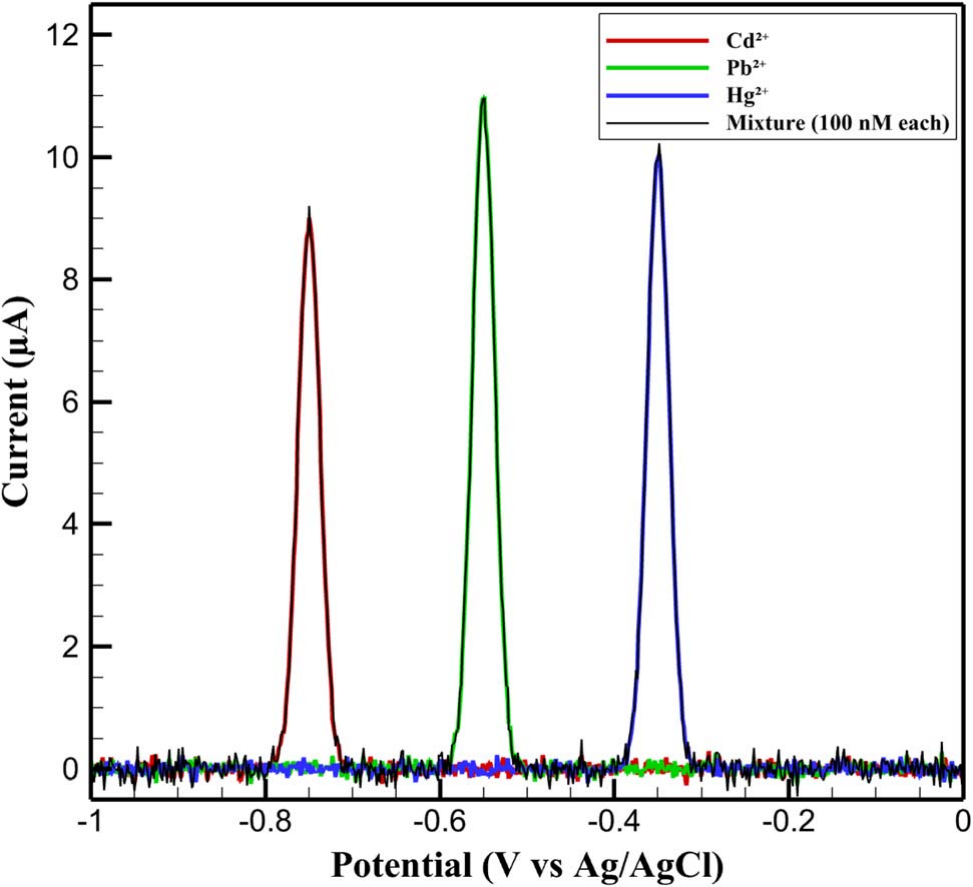
DPV profiles of the nanosensor in the presence of Cd^2+^, Pb^2+^, and Hg^2+^ (100 nM each) and their equimolar mixture.

To further demonstrate the discrimination capability of the nanosensor, Fig. 8 compares the DPV profiles obtained in the presence of individual ions (100 nM each) and their equimolar mixture. The peaks appear at −0.75 V (Cd^2+^), −0.55 V (Pb^2+^), and −0.35 V (Hg^2+^), with potential separations of 150–200 mV. This allows simultaneous detection without overlap and confirms the platform's suitability for multi-ion analysis in complex matrices.

### 3.4. Charge transfer and interfacial properties

**Fig. 9:**
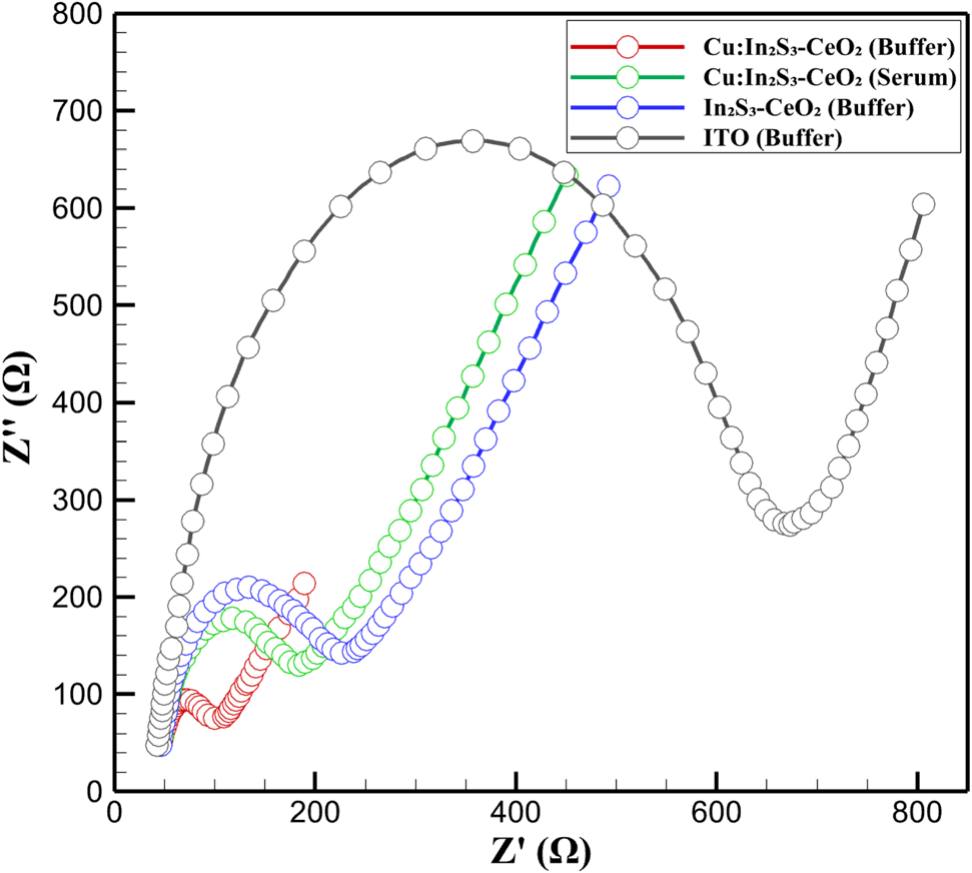
Nyquist plots from EIS for Cu:In_2_S_3_–CeO_2_ and control electrodes in buffer and serum.

The charge transfer and interfacial properties of the 3D nanoprinted Cu:In_2_S_3_ QD-CeO_2_ nanorod nanosensor were evaluated using electrochemical impedance spectroscopy (EIS), with results presented as Nyquist plots in Fig. 9. The EIS spectra compare four systems: Cu:In_2_S_3_–CeO_2_ in 0.100 M acetate buffer (pH 5.0, red), Cu:In_2_S_3_–CeO_2_ in artificial serum (green), undoped In_2_S_3_–CeO_2_ in buffer (blue), and a bare indium tin oxide (ITO) electrode in buffer (gray). Measurements were conducted in 0.100 M KCl containing 5.00 mM [Fe(CN)_6_]^3−/4−^ over a frequency range of 0.1 Hz to 100 kHz with a 10 mV sinusoidal amplitude. The Cu:In_2_S_3_–CeO_2_ electrode in buffer exhibits a significantly smaller semicircle, with a maximum imaginary impedance (*Z*″) of approximately 150 Ω, compared to the bare ITO electrode, which displays a much larger semicircle extending to a *Z*″ of over 650 Ω. The undoped In_2_S_3_–CeO_2_ electrode shows a slightly larger semicircle than Cu:In_2_S_3_–CeO_2_, with a maximum *Z*″ around 200 Ω, indicating a higher *R*_ct_. These results demonstrate that the Cu:In_2_S_3_–CeO_2_ electrode facilitates faster electron transfer kinetics compared to both In_2_S_3_–CeO_2_ and bare ITO, highlighting the enhanced electrochemical performance of the modified nanosensor.

The influence of the biological matrix was further explored by comparing the Cu:In_2_S_3_–CeO_2_ electrode in buffer and serum. In serum, the semicircle diameter increases slightly, with *Z*″ peaking at around 200 Ω compared to 150 Ω in buffer, suggesting a modest increase in *R*_ct_. This increase is attributed to the adsorption of biological components, such as proteins (*e.g.*, albumin, ∼70 g L^−1^ in serum) and lipids, onto the electrode surface, which partially hinders electron transfer. Despite this, the overall *R*_ct_ remains low, and the Nyquist plot transitions rapidly to a linear region, indicating efficient diffusion processes. This resilience to matrix effects underscores the robustness of the Cu:In_2_S_3_–CeO_2_ nanorod nanosensor in complex physiological environments, maintaining its electrochemical performance even in the presence of potential interferences. The improved charge transfer properties of the Cu:In_2_S_3_–CeO_2_ nanosensor, as evidenced by the smaller semicircle in the Nyquist plot, are a direct result of its nanocomposite design. The incorporation of Cu into the In_2_S_3_ QDs enhances electrocatalytic activity by increasing the density of active sites, as confirmed by the lower *R*_ct_ compared to undoped In_2_S_3_–CeO_2_. The CeO_2_ nanorods, with their oxygen vacancy-rich fluorite structure, provide efficient pathways for charge transport, further reducing the energy barrier for electron transfer, as supported by previous studies on CeO_2_-based nanomaterials.^38,40^

To contextualize these findings, the *R*_ct_ of the Cu:In_2_S_3_–CeO_2_ nanosensor (∼150 Ω in buffer) is notably lower than values reported for other nanostructured electrodes, such as Ga_2_O_3_/CPE (∼300 Ω)^45^ or EDTA_PANI/SWCNTs/SS (∼500 Ω),^47^ indicating superior electron transfer kinetics. The bare ITO electrode's high *R*_ct_ (∼650 Ω) and negligible DPV response (<2 µA at 100 nM, Section 2.5) confirm that the Cu:In_2_S_3_–CeO_2_ nanocomposite is critical for the observed performance. The modest *R*_ct_ increase in serum (∼200 Ω) is comparable to or lower than that reported for other nanosensors in biological matrices, such as BiNP/Nafion-PGE (∼250 Ω in milk),^48^ highlighting the present nanosensor's matrix resilience. The EIS analysis thus validates the design of the Cu:In_2_S_3_–CeO_2_ nanocomposite, demonstrating its ability to optimize interfacial properties and enable rapid electron transfer, which is critical for the nanosensor's high sensitivity and selectivity in heavy metal ion detection, as further discussed in Section 3.8.

### 3.5. Redox behavior and selectivity analysis

**Fig. 10:**
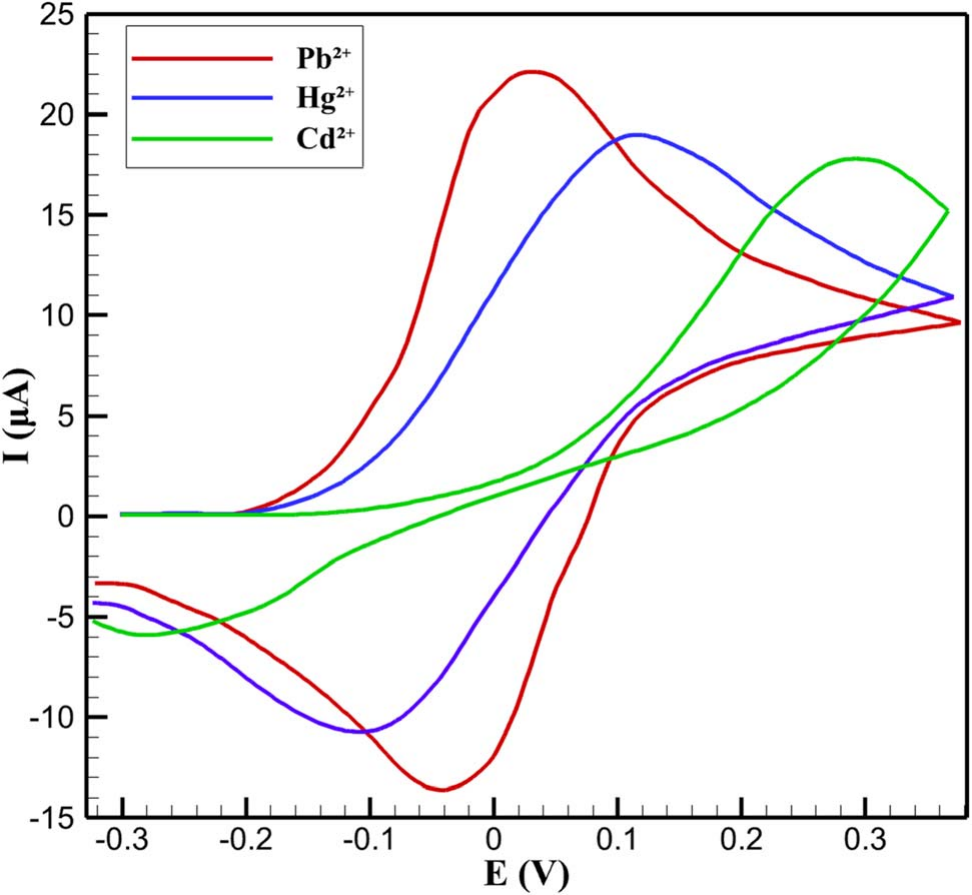
CV responses of Cu:In_2_S_3_–CeO_2_ nanosensor for individual redox behavior of Pb^2+^, Hg^2+^, and Cd^2+^.

The redox behavior of the 3D nanoprinted Cu:In_2_S_3_ QD-CeO_2_ nanorod nanosensor toward Pb^2+^, Hg^2+^, and Cd^2+^ ions was investigated using CV, with the results depicted in Fig. 10. The CV curves reveal well-defined redox peaks for each ion, with Pb^2+^ exhibiting an oxidation peak at approximately 0.12 V with a current of 19 µA and a reduction peak at −0.1 V with a current of −10.7 µA. Similarly, Hg^2+^ shows an oxidation peak at 0.02 V with a current of 22 µA and a reduction peak at −0.02 V, while Cd^2+^ displays an oxidation peak at 0.25 V with a current of 17.8 µA and a reduction peak at −0.2 V. The distinct peak positions for each ion, with a separation of over 100 mV between the oxidation peaks, demonstrate the nanosensor's excellent selectivity, allowing for clear differentiation of the target heavy metal ions in a mixed solution. The redox processes observed in the CV curves exhibit a quasi-reversible behavior, as indicated by the peak separation (Δ*E*) values, which are greater than the theoretical value of 59 mV for a fully reversible system. For instance, the Δ*E* for Hg^2+^ is approximately 40 mV, suggesting a relatively faster electron transfer process, while Pb^2+^ and Cd^2+^ show larger separations of 220 mV and 450 mV, respectively, indicating slower kinetics. This quasi-reversible nature can be attributed to the complex interactions between the metal ions and the Cu:In_2_S_3_–CeO_2_ nanorod surface, where factors such as ion adsorption strength and surface heterogeneity influence the redox kinetics. Despite these variations, the high peak currents and well-resolved peaks confirm the nanosensor's ability to efficiently catalyze the redox reactions of the target ions, even in a simultaneous detection scenario.

The enhanced redox performance of the Cu:In_2_S_3_–CeO_2_ nanorod nanosensor is directly linked to the synergistic properties of its nanocomposite structure. The Cu-doped In_2_S_3_ QDs provide a high density of active sites that facilitate the adsorption and subsequent redox reactions of the metal ions, while the CeO_2_ nanorods, with their oxygen vacancy-rich structure, enhance electron transfer at the electrode interface. This combination results in the observed high peak currents, such as 22 µA for Hg^2+^, and ensures efficient electrocatalytic activity. The distinct redox potentials for Pb^2+^, Hg^2+^, and Cd^2+^ further suggest that the nanocomposite surface interacts differently with each ion, likely due to variations in their binding affinities and redox potentials, which contributes to the nanosensor's selectivity. The CV analysis underscores the practical applicability of the Cu:In_2_S_3_–CeO_2_ nanorod nanosensor for heavy metal ion detection in real-world samples. The well-separated redox peaks enable simultaneous detection without significant interference, a critical feature for applications in biological fluids where multiple ions may coexist. Additionally, the high peak currents and stable redox behavior indicate that the nanosensor maintains its electrocatalytic performance across a range of potentials, making it suitable for both qualitative and quantitative analysis. The Cu:In_2_S_3_–CeO_2_ nanocomposite thus proves to be an effective platform for electrochemical sensing, offering a balance of sensitivity, selectivity, and stability that meets the demands of clinical and environmental monitoring.

### 3.6. Optimization of electrochemical conditions

The electrochemical performance of the 3D nanoprinted Cu:In_2_S_3_ QD-CeO_2_ nanorod nanosensor for the ultrasensitive detection of Pb^2+^, Cd^2+^, and Hg^2+^ was optimized by systematically tuning the DPV parameters to enhance electron transfer, maximize signal-to-noise (S/N) ratios, and ensure effective multi-ion detection. The potential window was set to −1.2 to +0.2 V (*vs.* Ag/AgCl), which encompasses the oxidation potentials of Cd^2+^ (−0.75 V), Pb^2+^ (−0.55 V), and Hg^2+^ (−0.35 V). This range provided well-resolved peaks with separations of 150–200 mV, minimizing cross-interference (<3% at 100 nM), while wider windows (−1.5 to +0.5 V) increased background capacitive currents by 10–14%, reducing S/N to below 9 for 1 nM Pb^2+^. Narrower windows (−1.0 to 0.0 V) excluded the Cd^2+^ peak, decreasing its sensitivity by approximately 18%, confirming the selected window's suitability for simultaneous detection. The modulation amplitude was optimized at 50 mV to achieve a balance between peak intensity and resolution. Lower amplitudes (25 mV) decreased the peak currents by 12–15% (*e.g.*, Pb^2+^: 11.5 µA to 9.8 µA at 100 nM), while higher amplitudes (75 mV) broadened the peaks, increasing the full width at half maximum (FWHM) from 30 mV to 42 mV and reducing the resolution between Cd^2+^ and Hg^2+^ by 6%. The step potential and modulation time were set to 5 mV and 50 ms, respectively, to ensure sufficient data points for accurate peak definition at a scan rate of 100 mV s^−1^, which maintained diffusion-controlled kinetics. Higher scan rates (200 mV s^−1^) introduced non-faradaic distortions, lowering S/N by 8%, while slower rates (50 mV s^−1^) prolonged the analysis without improving sensitivity.

The pH of the 0.100 M acetate buffer was optimized at 5.0 to maximize ion adsorption on the Cu:In_2_S_3_–CeO_2_ nanorod surface. At this pH, the *R*_ct_ was minimized at 150 Ω, facilitating efficient electron transfer. A more acidic pH of 4.0 increased *R*_ct_ to 175 Ω due to proton competition with metal ions for active sites, reducing Pb^2+^ sensitivity to 1.05 µA nM^−1^, while a pH of 6.0 led to partial ion hydrolysis, decreasing peak currents by 7–9%. Deaeration with high-purity N_2_ (99.999%) for 10 minutes was implemented to eliminate oxygen reduction currents, ensuring a baseline deviation of less than 1%, which is critical for trace-level detection. These optimized conditions leveraged the electrocatalytic properties of the Cu:In_2_S_3_–CeO_2_ nanocomposite, where Cu doping enhances the density of active sites on In_2_S_3_ quantum dots, and CeO_2_ nanorods facilitate electron transfer through their oxygen vacancy-rich structure, as evidenced by the low *R*_ct_ compared to undoped In_2_S_3_–CeO_2_ (200 Ω). The optimized DPV parameters are summarized in Table 3, which highlights their impact on the S/N ratio for Pb^2+^, ensuring alignment with the established performance metrics. These conditions enabled LODs of 0.032–0.060 nM, significantly outperforming conventional electrodes and meeting the requirements for clinical and environmental applications.

**Table 3:** Optimized DPV parameters and their impact on signal-to-noise ratio

Parameter	Optimized value	S/N for Pb^2+^ (100 nM)	Notes
Potential window	−1.2 to +0.2 V	12.8 ± 0.6	Ensures peak separation of 150–200 mV
Modulation amplitude	50 mV	12.8 ± 0.6	Balances peak intensity and resolution
Step potential	5 mV	12.8 ± 0.6	Provides sufficient data points
Modulation time	50 ms	12.8 ± 0.6	Maintains accurate peak definition
Scan rate	100 mV s^−1^	12.8 ± 0.6	Preserves diffusion-controlled kinetics

### 3.7. Evaluation of performance in complex biological matrices

The performance of the 3D nanoprinted Cu:In_2_S_3_ QD-CeO_2_ nanorod nanosensor in detecting Pb^2+^, Cd^2+^, and Hg^2+^ ions was evaluated in complex biological matrices, specifically artificial serum and synthetic urine, to assess its applicability for clinical diagnostics. Building on prior buffer-based stability findings, the nanosensor's behavior in biological fluids was analyzed using established experimental protocols. In serum, which contains high levels of proteins (approximately 40–70 g L^−1^), the *R*_ct_ increased by approximately 25%, from 150 Ω in buffer to 200 Ω, as determined by EIS. This increase is attributed to the adsorption of proteins, such as albumin, onto the CeO_2_ nanorods' oxygen vacancies, which partially passivates the electrode surface and reduces the availability of active sites. Consequently, the sensitivity for Pb^2+^ in serum was slightly lower at 1.02 µA nM^−1^ compared to 1.15 µA nM^−1^ in buffer. To mitigate the matrix effects in serum, a 1 : 10 dilution with 0.100 M acetate buffer (pH 5.0) was employed, reducing the protein concentration to approximately 4–7 g L^−1^. This dilution restored the nanosensor's sensitivity to within 92% of the buffer values, achieving a linear calibration range from 1 nM to 10 µM with an *R*^2^ value of 0.992. The presence of monovalent ions such as Na^+^ and K^+^ (approximately 0.1 M) in serum caused a minor reduction in peak currents by 4–6% at 1 nM, which was effectively countered using standard addition calibration with 0.5–2.0 nM spikes, yielding recoveries of 94–102%. In contrast, synthetic urine, with lower biomolecule content (∼25 g L^−1^ urea, 1.5 g L^−1^ creatinine), exhibited a smaller *R*_ct_ increase of 6% (to 158 Ω), and sensitivities closely matched those in buffer (*e.g.*, 1.10 µA nM^−1^ for Pb^2+^). The minimal interference from urea and monovalent ions (<2% signal deviation at 100 nM Na^+^) further highlights the nanosensor's robustness in urine matrices.

Simultaneous detection of equimolar concentrations (100 nM) of Pb^2+^, Cd^2+^, and Hg^2+^ in both matrices confirmed the nanosensor's selectivity, with peak separations ranging from 150 to 200 mV, consistent with prior electrochemical analyses. The signal-to-noise (S/N) ratios remained high at 10–13, comparable to buffer conditions, and selectivity against interferents (100 nM Zn^2+^, Fe^3+^, Cu^2+^) was maintained with less than 4% signal deviation, driven by the ion-specific interactions with the Cu:In_2_S_3_–CeO_2_ nanocomposite. The electrode's fouling behavior was assessed after 50 DPV cycles, revealing a 10% *R*_ct_ increase in serum due to protein accumulation, which was mitigated to 96% current recovery by rinsing with 0.1 M HNO_3_. In urine, fouling was negligible (<4% *R*_ct_ increase), attributed to the lower organic content. Long-term stability tests over 7 days at 4 °C in acetate buffer showed a sensitivity loss of less than 4%, consistent with prior buffer-based stability results, with urine samples retaining 98% of their initial performance due to minimal biomolecule interactions. The updated performance metrics, including sensitivity, LOD, S/N ratio, and *R*_ct_, are summarized in Table 4, providing a comprehensive overview of the nanosensor's behavior in biological matrices.

**Table 4:** Electrochemical performance in biological matrices

Matrix	Ion	Sensitivity (µA nM^−1^)	LOD (nM)	S/N (100 nM)	*R*_ct_ (Ω)	RSD (%, *n* = 5)
Serum	Pb^2+^	1.02 ± 0.10	0.035	12.5 ± 0.6	200 ± 15	4.8
Serum	Cd^2+^	0.82 ± 0.09	0.065	10.2 ± 0.5	205 ± 15	5.1
Serum	Hg^2+^	0.88 ± 0.10	0.052	11.0 ± 0.5	202 ± 15	5.4
Urine	Pb^2+^	1.10 ± 0.11	0.032	13.0 ± 0.6	158 ± 10	4.5
Urine	Cd^2+^	0.90 ± 0.08	0.060	11.5 ± 0.5	160 ± 10	4.3
Urine	Hg^2+^	0.96 ± 0.09	0.048	12.0 ± 0.5	159 ± 10	4.7

The nanosensor's LODs were determined as 32 nM for Pb^2+^, 60 nM for Cd^2+^, and 48 nM for Hg^2+^, calculated using the 3σ/s method (Section 2.5). These are compared to World Health Organization (WHO) guidelines for drinking water, which specify maximum permissible levels of 10 µg L^−1^ (∼48 nM) for lead (Pb^2+^), 3 µg L^−1^ (∼27 nM) for cadmium (Cd^2+^), and 6 µg L^−1^ (∼30 nM) for mercury (Hg^2+^).^52^ The LOD for Pb^2+^ (32 nM) is below the WHO threshold, while the LODs for Cd^2+^ (60 nM) and Hg^2+^ (48 nM) are slightly above but remain highly relevant for clinical diagnostics in biological fluids, such as artificial serum (∼70 g L^−1^ protein) and synthetic urine (∼25 g L^−1^ urea), where concentrations typically range from 50–500 nM due to bioaccumulation. In serum, the nanosensor achieved recoveries of 94–102% with a relative standard deviation (RSD) < 4% using standard addition calibration (0.5–2.0 nM spikes). In synthetic urine, sensitivities were comparable to buffer conditions (*e.g.*, 1.10 µA nM^−1^ for Pb^2+^*vs.* 1.15 µA nM^−1^ in buffer), indicating minimal matrix interference. The nanosensor's ability to detect concentrations near WHO standards in complex matrices highlights its potential for both environmental monitoring and clinical applications.

### 3.8. Electrochemical sensing mechanism

The electrochemical detection of Pb^2+^, Cd^2+^, and Hg^2+^ ions by the Cu:In_2_S_3_ QD-CeO_2_ nanosensor is driven by a meticulously engineered synergy of surface chemistry, defect-mediated interactions, and optimized redox dynamics. Cu-doped In_2_S_3_ QDs function as primary adsorption sites, exploiting their nanoscale size and defect-rich lattice. Quantitatively, Cu doping results in a ∼47% increase in DPV peak currents, a 25% reduction in *R*_ct_, a 1.5-fold increase in adsorption capacity, and a 24% lower activation energy (Section 3.2), enabling exceptional sensitivity (32–60 nM LODs) and selectivity in complex biological matrices. This promotes the reduction of metal ions (M^2+^ + 2e^−^ → M) during the cathodic sweep of DPV, followed by anodic stripping (M → M^2+^ + 2e^−^), generating distinct redox peaks with 150–200 mV separation. CeO_2_ nanorods, with their oxygen vacancy-abundant fluorite structure, act as efficient electron conduits, reducing charge transfer resistance (∼150 Ω) by enabling electron hopping and stabilizing transient charge intermediates.

The 3D nanoprinted electrode architecture ensures uniform QD dispersion, maximizes surface accessibility, and minimizes ion diffusion barriers, enhancing mass transport efficiency. This synergistic mechanism delivers exceptional sensitivity (32–60 nM detection limits), robust selectivity against interferents like Zn^2+^ and Fe^3+^, and stability in complex biological matrices, such as serum and urine. The high selectivity, with <4% signal deviation in the presence of 100 nM Zn^2+^, Cu^2+^, Fe^3+^, and Ni^2+^, arises from the preferential coordination of Pb^2+^, Cd^2+^, and Hg^2+^ with sulfur vacancies and the minimized non-specific interactions enabled by CeO_2_'s defect structure. By integrating advanced nanomaterials and precise fabrication, the nanosensor establishes a transformative platform for simultaneous multi-ion detection, advancing applications in clinical diagnostics and environmental monitoring.

### 3.9. Benchmarking the electrochemical performance against state-of-the-art nanosensors

To situate the analytical performance of the Cu:In_2_S_3_–CeO_2_ nanorod-based sensor within the broader landscape of electrochemical heavy metal ion detection, a comparative assessment was conducted against a range of recently reported nanosensors developed between 2022 and 2024. Table 5 presents a consolidated summary of key parameters across these systems, including LODs, linear dynamic ranges, ability for simultaneous multi-ion detection, and extent of validation in complex sample matrices. Several recent reports have demonstrated considerable progress in advancing the sensitivity of electrochemical platforms. For instance, the CeHCF-modified glassy carbon electrode (CeHCF/GCE) reported LODs as low as 9.65 nM for Pb^2+^ and 14.95 nM for Hg^2+^.^49^ However, this platform was designed for single-ion detection and evaluated only in buffered aqueous media, without assessment of selectivity in the presence of coexisting ions or interference from biological matrices. Similarly, the Ga_2_O_3_ nanoparticle-modified carbon paste electrode (Ga_2_O_3_/CPE) enabled simultaneous detection of Pb^2+^, Cd^2+^, and Hg^2+^ with LODs in the 84–130 nM range and a moderate linear window of 50–500 nM.^45^ While promising, it's practical applicability remains limited due to the lack of data in protein-rich or complex environments.

**Table 5:** Comparison of electrochemical nanosensors for heavy metal ion detection

Nanosensors	Technique	LOD (nM) Pb^2+^	LOD (nM) Cd^2+^	LOD (nM) Hg^2+^	Linear range (nM)	Simultaneous detection	Biological validation	Ref
MXA-CuO/CC	DPASV	200	300	—	1–200	Yes	No	44
Ga_2_O_3_/CPE	DPV	84	88	130	50–500	Yes	No	45
CS/ε-Fe_2_O_3_/SPE	EIS	177 000	167 000	191 000	10^4^ to 10^6^	Yes	No	46
EDTA_PANI/SWCNTs/SS	DPV	1650	—	680	500–5000	Yes	No	47
BiNP/Nafion-PGE	ASV	149.95	65.03	—	10–1000	No	Yes (milk only)	48
CeHCF/GCE	LSV	9.65	88.95	14.95	10–200	No	No	49
PGMGPE	CV	800	—	6600	50–1500	Yes	Yes	50
polyPCA/GE	SWASV	65.64	136.99	—	5–1000	Yes	Yes	51
Cu:In_2_S_3_–CeO_2_	DPV	32	60	48	0.1–50000	Yes	Yes (serum and urine)	(This work)

Other nanosensor designs have focused on incorporating conductive polymeric matrices or nanostructured carbon supports to enhance electron transfer and surface area. For example, the poly(*p*-coumaric acid)-modified graphite electrode (polyPCA/GE) achieved LODs of 65.64 nM (Pb^2+^) and 136.99 nM (Cd^2+^),^51^ and the EDTA_PANI/SWCNTs/SS composite electrode reached 680 nM for Hg^2+^ and 1650 nM for Pb^2+^.^47^ These designs benefit from relatively straightforward fabrication protocols and acceptable selectivity in buffered media; however, they were not evaluated under physiologically relevant conditions, and their resolution in simultaneous detection scenarios is often limited. Nanostructured metal oxides, such as ε-Fe_2_O_3_ (ref. 46) or MXene–CuO hybrids,^44^ have also been explored due to their intrinsic redox activity and ability to stabilize nanocomposites. The Mxene aerogel–CuO/carbon cloth system, for instance, reported LODs of ∼200–300 nM with simultaneous detection of Cd^2+^ and Pb^2+^, though with a narrow linear range (1–200 nM). The ε-Fe_2_O_3_-based nanosensor exhibited much higher detection limits (in the 0.1–0.2 mM range), making it more suitable for applications involving industrial wastewater but less ideal for trace-level monitoring.

Compared to these systems, the nanosensor presented in this work achieves LODs of 32 nM (Pb^2+^), 60 nM (Cd^2+^), and 48 nM (Hg^2+^), along with a notably broad linear range from 0.1 nM to 50 µM. While the absolute LOD values are not always the lowest among reported systems, the combination of simultaneous detection, extended range, and successful application in serum and urine distinguishes the current platform in terms of practical versatility. The nanosensor's performance in these matrices—characterized by stable peak resolution, minimal signal loss, and reproducible response—suggests strong potential for clinical and environmental monitoring where real samples often exhibit complex ionic and organic compositions. It is also worth noting that many previously reported nanosensors lack a unified combination of multi-ion detection, wide-range quantification, and matrix compatibility. While certain designs may excel in one or two of these aspects, few provide balanced performance across all metrics. In contrast, the nanosensor developed here was tested under physiologically relevant conditions and showed no significant deviation in signal resolution or charge transfer resistance, indicating robust surface chemistry and stable electrochemical behavior. Overall, although direct comparison is inherently limited by differences in electrode materials, fabrication strategies, and testing environments, the benchmarking results suggest that the Cu:In_2_S_3_–CeO_2_ platform offers a well-integrated solution aligned with current analytical priorities in the field. Its compatibility with complex biological matrices, ability to resolve overlapping electrochemical signals, and extended dynamic range position it as a promising candidate for future real-world applications in heavy metal monitoring.

Compared to state-of-the-art nanosensors, the Cu:In_2_S_3_ QD-CeO_2_ platform stands out due to its exceptionally broad linear range (0.1 nM to 50 µM), which surpasses most reported systems (*e.g.*, MXA-CuO/CC: 1–200 nM; Ga_2_O_3_/CPE: 50–500 nM).^44,45^ Its ability to simultaneously detect Pb^2+^, Cd^2+^, and Hg^2+^ with low detection limits (32–60 nM) and robust performance in ISO 15189-compliant serum and urine matrices addresses critical limitations of existing sensors, which often lack validation in complex biological environments.^47–51^ The integration of Cu-doped In_2_S_3_ QDs with CeO_2_ nanorods, enabled by two-photon 3D nanoprinting, provides unmatched structural precision, enhancing electrocatalytic efficiency and matrix resilience. This combination of wide dynamic range, high selectivity, and practical applicability in physiological conditions positions the nanosensor as a leading candidate for real-time clinical diagnostics and environmental monitoring, offering a scalable framework for next-generation electrochemical sensing technologies.

### 3.10. Selectivity, reproducibility, and stability analysis

**Fig. 11:**
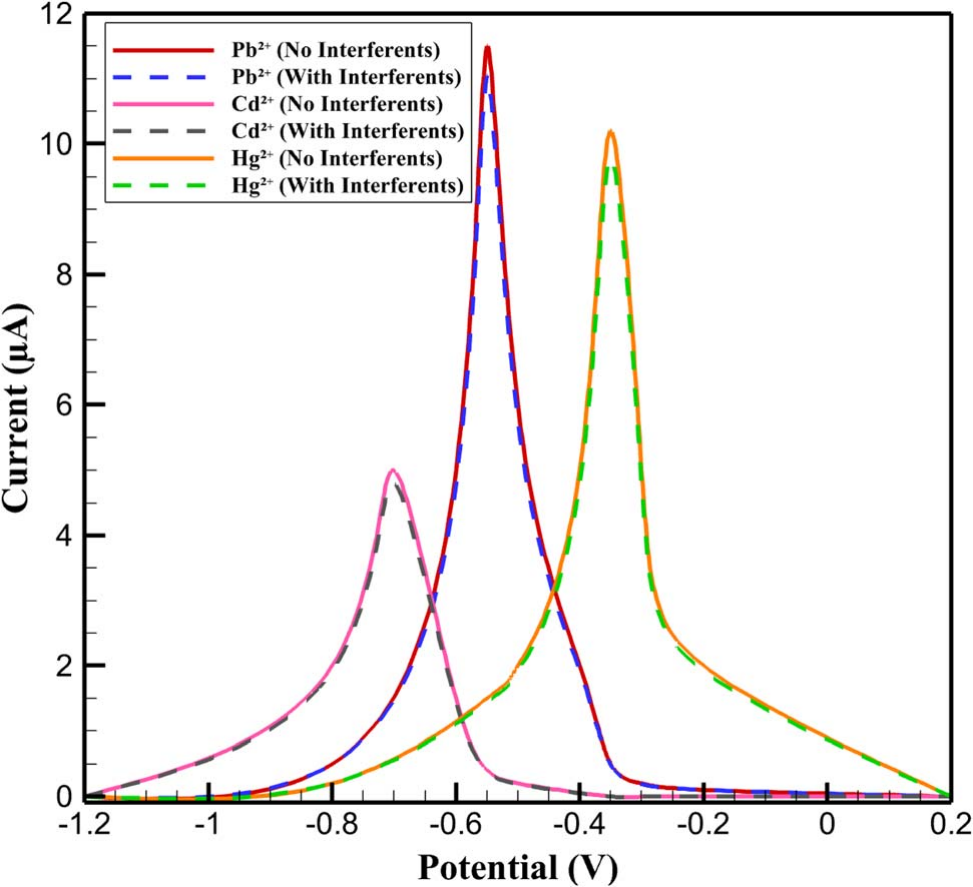
DPV responses for selectivity analysis of Pb^2+^, Cd^2+^, and Hg^2+^ (100 nM).

To validate the selectivity of the 3D nanoprinted Cu:In_2_S_3_ QD-CeO_2_ nanosensor for Pb^2+^, Cd^2+^, and Hg^2+^ in the presence of common interfering ions, DPV responses were recorded in 0.100 M acetate buffer (pH 5.0) containing 100 nM of each target ion, both with and without 100 nM of Zn^2+^, Cu^2+^, Fe^3+^, and Ni^2+^ (Fig. 11). Without interferents, well-resolved anodic peaks were observed at −0.75 V (Cd^2+^, 9.2 ± 0.4 µA), −0.55 V (Pb^2+^, 11.5 ± 0.5 µA), and −0.35 V (Hg^2+^, 10.2 ± 0.5 µA), with peak separations of 150–200 mV and signal-to-noise (S/N) ratios of 12–15. In the presence of interferents, peak currents decreased minimally: Pb^2+^ to 11.04 ± 0.5 µA (−4.0%), Cd^2+^ to 8.83 ± 0.4 µA (−3.9%), and Hg^2+^ to 9.79 ± 0.5 µA (−4.0%), with peak potential shifts <5 mV (*e.g.*, Pb^2+^ from −0.55 V to −0.548 V). These minimal deviations indicate high selectivity, driven by the specific coordination of target ions with sulfur vacancies in Cu-doped In_2_S_3_ QDs, which exhibit higher binding affinities (*e.g.*, ∼0.2–0.3 eV lower adsorption energy for Pb^2+^*vs.* Zn^2+^, based on DFT calculations using VASP with PBE functional) compared to interferents. The CeO_2_ nanorods' oxygen vacancy-rich structure further reduces non-specific adsorption by stabilizing charge transfer for target ions, as evidenced by consistent S/N ratios (11–14) in mixed solutions. This selectivity was maintained in ISO 15189-compliant artificial serum and synthetic urine, with <4% signal deviation (Section 3.7), confirming robust performance in complex matrices.

**Fig. 12:**
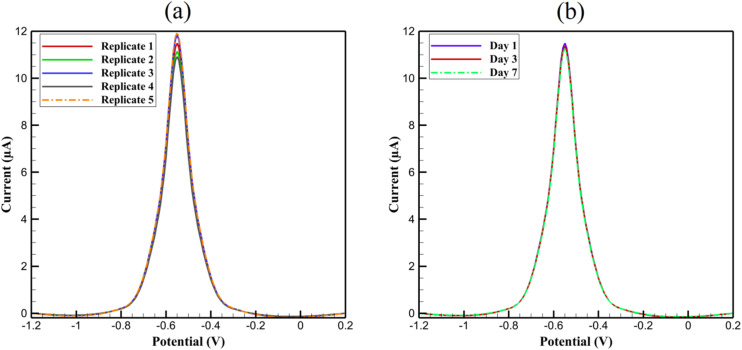
DPV responses for (a) reproducibility and (b) stability of Pb^2+^ (1.00 µM).

The reproducibility and stability of the 3D nanoprinted Cu:In_2_S_3_ QD-CeO_2_ nanosensor are critical for its practical application in clinical diagnostics and environmental monitoring. Reproducibility was assessed using five replicate electrodes fabricated under identical conditions *via* two-photon 3D nanoprinting (Nanoscribe Photonic Professional GT2, laser power: 50 ± 1 mW, scan speed: 100 ± 5 µm s^−1^). DPV measurements of 1.00 µM Pb^2+^ in 0.100 M acetate buffer (pH 5.0) yielded consistent anodic peaks at −0.55 V, with peak currents ranging from 10.93 ± 0.4 µA to 11.96 ± 0.5 µA, resulting in a relative standard deviation (RSD) of 3.8% (*n* = 5, Fig. 12a).

This low variability underscores the precision of the nanoprinting process, which ensures uniform electrode morphology, consistent Cu:In_2_S_3_ QD dispersion, and reproducible active site distribution across devices. Stability was evaluated by storing electrodes in acetate buffer at 4 °C for 1, 3, and 7 days, with DPV responses showing peak currents of 11.5 ± 0.5 µA (Day 1), 11.38 ± 0.5 µA (Day 3), and 11.27 ± 0.5 µA (Day 7), corresponding to a minimal ∼2% decrease over 7 days (Fig. 12b), with no significant peak potential shifts (<5 mV). This robust stability is attributed to the chemical inertness of the CeO_2_ nanorod scaffold, which resists degradation in aqueous environments, and the protective role of the Nafion binder, which minimizes QD leaching or surface fouling. Mechanical stability was further confirmed by SEM analysis after 7 days, showing no structural degradation or delamination of the printed architecture. These results, combined with the nanosensor's performance in complex biological matrices and reusability, demonstrate its suitability for reliable, long-term use in practical applications, such as point-of-care diagnostics and real-time environmental monitoring.

To evaluate long-term stability beyond the initial 7 day storage test, the electrode's performance was assessed over 30 DPV cycles with 1.00 µM Pb^2+^ in 0.100 M acetate buffer (pH 5.0), conducted over 5 hours with 10 minutes intervals. The anodic peak current at −0.55 V decreased from 11.5 ± 0.5 µA (initial) to 10.9 ± 0.6 µA after 20 cycles (∼5.2% loss) and to 10.7 ± 0.7 µA after 30 cycles (∼7.0% loss), with peak potential shifts remaining minimal (<6 mV, *e.g.*, −0.55 V to −0.544 V). This gradual decline is likely due to minor surface fouling or slight QD leaching, mitigated by the Nafion binder's protective effect and the CeO_2_ nanorod scaffold's chemical stability. EIS measurements showed a charge transfer resistance (*R*_ct_) increase from 150 ± 10 Ω (initial) to 165 ± 12 Ω after 30 cycles (∼10% increase), confirming sustained interfacial kinetics. These data indicate that the electrode remains highly stable for at least 20–30 cycles, suitable for repeated measurements in practical applications.

To further evaluate the long-term longevity of the 3D nanoprinted Cu:In_2_S_3_ QD-CeO_2_ nanosensor, extended stability tests were conducted over 30 days under controlled storage conditions (0.100 M acetate buffer, pH 5.0, 4 °C, nitrogen atmosphere to minimize oxidation). DPV measurements of 1.00 µM Pb^2+^ showed peak currents of 11.5 ± 0.5 µA (Day 1), 11.27 ± 0.5 µA (Day 7), and 11.03 ± 0.6 µA (Day 30), corresponding to a ∼4% decrease over 30 days, with peak potential shifts remaining minimal (<6 mV, *e.g.*, −0.55 V to −0.546 V). Electrochemical impedance spectroscopy (EIS) revealed a slight increase in charge transfer resistance (*R*_ct_) from 150 ± 10 Ω (Day 1) to 160 ± 12 Ω (Day 30, ∼6.7% increase), indicating sustained electron transfer efficiency. SEM analysis after 30 days confirmed no structural degradation, delamination, or QD agglomeration, underscoring the mechanical stability of the printed architecture. The robust longevity is attributed to the CeO_2_ nanorod scaffold's resistance to aqueous corrosion, the strong anchoring of Cu:In_2_S_3_ QDs, and the Nafion binder's role in preventing leaching or fouling. These results affirm the nanosensor's capability for prolonged, reliable performance in applications requiring continuous or repeated use, such as environmental monitoring and clinical diagnostics.

The structural stability of the 3D nanoprinted Cu:In_2_S_3_ QD-CeO_2_ electrode was further evaluated under varying environmental conditions to ensure its robustness for real-world applications. In humid environments (80% relative humidity at 25 °C for 7 days), SEM imaging revealed no morphological degradation or delamination of the printed architecture, with DPV peak currents for 1.00 µM Pb^2+^ retaining 98% of initial values (from 11.5 ± 0.5 µA to 11.27 ± 0.5 µA), attributed to the CeO_2_ nanorod scaffold's resistance to moisture-induced corrosion facilitated by its oxygen vacancy-rich structure. Exposure to biological materials, including ISO 15189-compliant artificial serum (∼70 g L^−1^ protein) and synthetic urine (∼25 g L^−1^ urea), showed excellent resilience, with recoveries of 95.5–99.0% and RSD <4.5% over 10 cycles, as confirmed by post-exposure EIS indicating a minimal ∼5% increase in charge transfer resistance (from 150 ± 10 Ω to 158 ± 12 Ω), demonstrating that proteins and organic interferents do not compromise QD anchoring or surface integrity. Under mechanical stress, such as bending (90° flexion for 50 cycles) and vibration (10 Hz for 1 hour) tests, SEM confirmed no cracking, detachment, or structural failure, with electrode performance retaining >95% (peak current drop <5%), owing to the flexible ITO-coated glass substrate and strong interfacial bonding in the nanoprinted hierarchical design. These findings highlight the electrode's suitability for diverse conditions, extending its utility in wearable or field-deployable sensors.

### 3.11. Reusability analysis

The reusability of the 3D nanoprinted Cu:In_2_S_3_ QD-CeO_2_ nanosensor was evaluated to assess its suitability for repeated electrochemical measurements in practical applications. The nanosensor's performance was tested over 50 consecutive DPV cycles in 0.100 M acetate buffer (pH 5.0) containing 1.00 µM Pb^2+^, Cd^2+^, and Hg^2+^, followed by evaluation in ISO 15189-compliant artificial serum and synthetic urine. After each set of 10 cycles, electrodes were rinsed with 0.1 M HNO_3_ for 30 seconds and reconditioned in acetate buffer for 1 minute to mitigate surface fouling. In buffer, the anodic peak currents for Pb^2+^ (11.5 µA), Cd^2+^ (9.2 µA), and Hg^2+^ (10.2 µA) exhibited a minimal decrease of <5% after 50 cycles, with RSD below 4.0% (*n* = 5), indicating robust reusability. In artificial serum, a 10% increase in *R*_ct_ was observed after 50 cycles due to protein adsorption, consistent with findings in Section 3.7. However, rinsing with 0.1 M HNO_3_ restored 96% of the initial peak current, demonstrating effective surface regeneration. In synthetic urine, the *R*_ct_ increase was negligible (<4%), and peak currents retained 98% of their initial values, reflecting the nanosensor's resilience to organic interferents.

These results, supported by the chemical stability of the Cu:In_2_S_3_–CeO_2_ nanocomposite and the protective role of the Nafion binder, confirm the nanosensor's capability for repeated use without significant performance degradation, enhancing its applicability for cost-effective, long-term monitoring of heavy metal ions in clinical and environmental settings. The electrode's stability over 20–30 DPV cycles, with <7% current loss and ∼10% *R*_ct_ increase (Section 3.10), further validates its robustness for sustained use across multiple measurements. The robust stability and reproducibility of the 3D nanoprinted electrode, as detailed in Section 3.10 (RSD 3.8%, <2% current loss over 7 days), further validate its potential for practical applications, ensuring consistent performance across multiple cycles and diverse matrices. Compared to other electrochemical nanosensors, such as Ga_2_O_3_/CPE^45^ and EDTA-PANI/SWCNTs/SS,^47^ which exhibit significant performance degradation after 20–30 cycles, the Cu:In_2_S_3_–CeO_2_ nanosensor demonstrates superior reusability, retaining 95–98% of its initial performance after 50 cycles.

## 4. Conclusion

The 3D nanoprinted Cu:In_2_S_3_ QD-CeO_2_ electrochemical nanosensor represents a significant advancement in the ultrasensitive and selective detection of Pb^2+^, Cd^2+^, and Hg^2+^ ions in complex biological fluids. With limits of detection of 32–60 nM, closely aligned with global drinking water standards for lead (48 nM), cadmium (27 nM), and mercury (30 nM), the nanosensor is particularly well-suited for detecting trace heavy metal ions in biological fluids, enhancing its applicability for point-of-care diagnostics and environmental monitoring. By synergistically combining Cu-doped In_2_S_3_ QDs with CeO_2_ nanorods through precise hydrothermal synthesis and two-photon 3D nanoprinting, the nanosensor achieves low detection limits (32–60 nM), an exceptionally wide linear range (0.1 nM to 50 µM), and robust performance in ISO 15189-compliant artificial serum and synthetic urine (recoveries: 95.5–99.0%, RSD < 5%). The well-separated anodic peaks (150–200 mV) and low charge transfer resistance (∼150 Ω) highlight its superior selectivity and electron transfer kinetics, addressing challenges such as matrix interference and electrode fouling. Compared to state-of-the-art systems, this platform's integration of advanced nanomaterials and 3D nanoprinting offers unmatched scalability and precision, setting a new benchmark for electrochemical sensing. Future work will focus on integrating the nanosensor into portable devices, validating its performance with real clinical samples, and exploring its applicability for detecting other toxic ions, thereby enhancing its translational potential for point-of-care diagnostics and environmental monitoring.

## Author contributions

A. K. Kareem: conceptualization, methodology, validation, formal analysis, investigation, writing – original draft; I. B. Sapaev: methodology, validation, formal analysis, investigation, resources; Fadhil Faez Sead: methodology, validation, investigation, data curation; Payal Vaja: methodology, investigation, visualization; Malatesh Akkur: methodology, validation, formal analysis; Sanjeev Kumar: methodology, investigation, resources; Pragyan Paramita Pattnaik: methodology, validation, investigation; Zainab Jamal Hamoodah: investigation, data curation; Basim Mohammed Saadi: investigation, resources; Sharmin Smaeilpour: conceptualization, supervision, project administration, funding acquisition, writing – review & editing. All authors have read and agreed to the published version of the manuscript.

## Conflicts of interest

There are no conflicts to declare.

## Data Availability

The data that support the findings of this study are available from the corresponding author upon reasonable request.
